# Linkerability of Protein Ligands: Insights From Cocrystal Structures and Implications for DNA‐Encoded Libraries

**DOI:** 10.1002/minf.70045

**Published:** 2026-08-02

**Authors:** Raphael M. Franzini

**Affiliations:** ^1^ Department of Medicinal Chemistry University of Utah Salt Lake City Utah USA

**Keywords:** combinatorial chemistry, DNA‐encoded library, linker, structure–activity relationships, surface accessibility

## Abstract

Linkers play a central role in many areas of medicinal chemistry, including proximity inducers, small‐molecule conjugates, and DNA‐encoded libraries. However, little is known about the accessibility of molecules to linker attachment when bound to proteins. Here, we analyze linker accessibility across protein–ligand complexes in cocrystal structures. A computational workflow was developed to evaluate the linkerability of modifiable positions on molecules based on solvent accessibility, local steric space for introduction of a linker atom, and the geometry of solvent‐directed escape paths approximated as conical frustums. Analysis of 8,228 protein–ligand cocrystal structures with 131 431 modifiable positions shows that approximately 22% of positions can accommodate linkers without significant geometric restriction. Limited linkerability of positions influences DEL data and may confound efforts to use such data for lead prediction.

## Graphical abstract

Linkers play a central role in medicinal chemistry but can interfere with protein binding of small molecules. This study analyzes linkerability of protein ligands and explores how limited accessibility impacts the interpretation of DNA‐encoded library data and their use in machine learning for predicting active compounds.

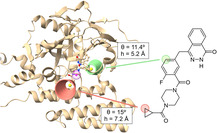

## 1 Introduction

Linkers are becoming increasingly important in medicinal chemistry. One reason for this trend is the emergence of therapeutic modalities based on inducing proximity [1, 2]. The success of proteolysis‐targeting chimeras (PROTACs) for targeted protein degradation exemplifies this class of molecules [3], and numerous other engagers have been developed that induce processes such as RNA degradation [4], protein stabilization [5], phosphorylation [6], changes in intracellular localization [7], and antibody recruitment [8]. Linkers also play a central role in small‐molecule‐based delivery of radionuclides and cytotoxic agents [9]. In addition, linkers are at the core of discovery technologies such as DNA‐encoded libraries (DELs), in which small molecules are attached to DNA barcodes that enable affinity‐based selection [10, 11]. DELs have yielded numerous hits [12], and a recent retrospective analysis by researchers at Roche and Genentech identified DELs as a primary source of developable leads in their pipeline [13].

Whereas traditional DEL workflows rely on identifying enriched hits followed by their synthesis and experimental validation, an alternative paradigm has recently emerged in which DEL results are used as datasets for machine learning (ML) approaches to predict suitable lead molecules [14, 15]. Numerous studies have demonstrated the feasibility of this strategy [15, 16, 17, 18, 19, 20, 21, 22, 23, 24, 25, 26, 27, 28]; however, computationally predicted leads often exhibit lower potency than direct library hits, and false positives remain common, indicating that further methodology improvements are required. A better understanding of the characteristics and limitations determinants of DEL data will therefore be critical for advancing this approach [29].

**FIGURE 1:**
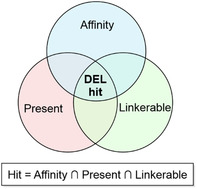
To appear as a hit, a DEL compound must (i) bind the target with high affinity, (ii) be present in the encoded library, and (iii) tolerate linker attachment at the selected position. The influence of linker attachment on DEL data is analyzed in this study.

For a DEL compound to emerge as a hit, three conditions must be met: (1) the molecule must bind the target with sufficient affinity, (2) the molecule must be physically present in the library, and (3) the attached linker must be compatible with binding to the protein (Figure 1). Predicting target affinity of molecules from DEL data is the goal of ML‐based DEL analysis. The problem of heterogeneous library composition has been recognized as a major confounding factor [30], and efforts have been undertaken to develop models that correct for it [16, 17]. In contrast, the influence of linkers is typically neglected. While solvent accessibility and steric effects are well recognized in individual structure‐based design efforts, there has been no systematic, large‐scale quantification of linkerability across protein–ligand complexes, nor a framework to relate these structural constraints to DEL outcomes.

As one of the few studies addressing linker effects in DELs, we recently examined the activity of compounds classified as nonhits and found that false negatives are widespread, at least in part because of linker interference [31]. However, the generality of this effect remains unclear.

Ideally, this question would be addressed through the analysis of numerous DEL selections across multiple targets to obtain a global understanding of linker effects. However, such a study would require prohibitive resources. The present work therefore approaches the problem from the reverse perspective, examining cocrystal structures of proteins bound to small‐molecule ligands and evaluating the fraction of ligand positions that are amenable to linker attachment. This analysis explores linkerability, defined as the ability of a position to accommodate a linker without a substantial decrease in binding energy or major changes to the protein–ligand binding geometry. It evaluates this by sequentially assessing solvent accessibility, local steric space, and the geometry of solvent‐directed surface cones. Analysis of 8228 protein–ligand complexes indicates that linker attachment is structurally restricted for the majority of ligand atoms. About ~22% of modifiable positions are predicted to accommodate unrestricted linker attachment. Together, these analyses provide a global view of the linkerability of protein‐bound small molecules and offer insight into how linker effects influence DEL selection outcomes.

## 2 Results and Discussion

### 2.1 Geometric Accessibility of Positions on Protein‐Bound Molecules

**FIGURE 2:**
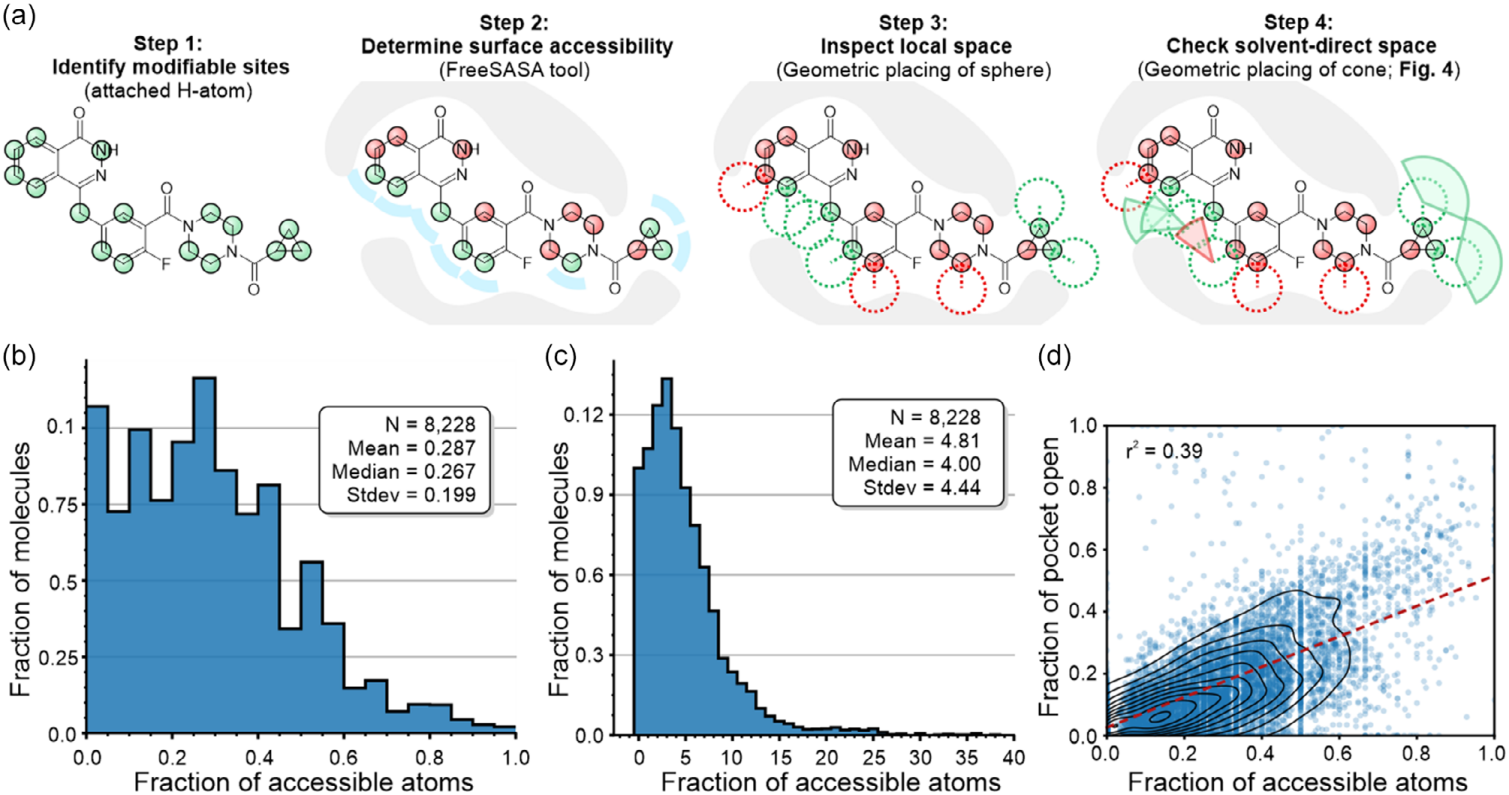
Geometric accessibility of positions on protein‐bound ligands. (a) Workflow and criteria used to determine accessibility based on the availability of a replaceable hydrogen, solvent accessible surface area, sufficient space for introduction of the first linker atom and an available path to the solvent. (b) Distribution of the fraction of modifiable positions on protein ligands that meet the two criteria of accessibility across 8228 protein–ligand complexes (steps 1–3). (c) Distribution of the number of accessible positions on protein ligands across 8228 protein–ligand complexes. (d) Relationship between the fraction of accessible atoms and the openness of the binding pocket.

As the first step, the geometric accessibility of positions on protein–bound ligands was evaluated. The criteria for accessibility of a position were (a) to have a replaceable hydrogen, (b) to have a solvent‐accessible surface area, and (c) to have space to accommodate the first atom of the linker chain; in a second step (d) the path to solvent was analyzed to gain further insights into linkerability (Figure 2a). The Python tool FreeSASA provided information on solvent‐accessible surface area [32], and a Python code was developed to use local molecular geometry to determine the position of the first linker atom and to test for clashes with the protein (see Supporting Information for details).

Accessible positions were identified across 8228 cocrystal structures randomly selected from the Protein Data Bank with filters to remove molecules irrelevant to the analysis (e.g., buffer molecules, fatty acids, and cofactors). In total, 131 431 modifiable atoms were identified and analyzed.

Of the identified modifiable atoms, 39 497 (30.1%) met the accessibility criteria. Analysis of the fraction of accessible positions on a per‐molecule basis (Figure 2b), revealed that molecules have a mean fraction of accessible positions of 0.287 (standard deviation = 0.199, median = 0.267). Correspondingly, molecules have on average 4.81 positions that are accessible with a median of 4 per molecule (Figure 2c). Approximately 10% of molecules lacked any accessible positions. Several reasons can account for the lack of accessible positions, including molecules at protein–protein interfaces, obstruction by other molecules and possessing only solvent‐exposed atoms lacking a replaceable hydrogen. A subset of molecules exhibited a very high proportion of accessible positions, corresponding to compounds present in the crystallization solution (e.g., buffer molecules, surfactants) that had escaped the filtering step. In conclusion, approximately 30% of modifiable positions in a typical protein ligand are accessible to the introduction of a linker.

This analysis relies on protein–ligand cocrystal structures, which are unavailable for many proteins. It was therefore tested whether global accessibility could be estimated from the geometric characteristics of the binding pockets. Plotting the fraction of accessible positions in ligands against the fraction of open surface area of the corresponding pocket resulted in a rough correlation (*r* ^2^ = 0.395; 95% confidence interval 0.377–0.411; Figure 2d). These results suggest that linker accessibility can be estimated from the properties of protein binding pockets even in the absence of structural information about bound small molecules.

### 2.2 Geometric Distribution of Accessible Position in Molecules

**FIGURE 3:**
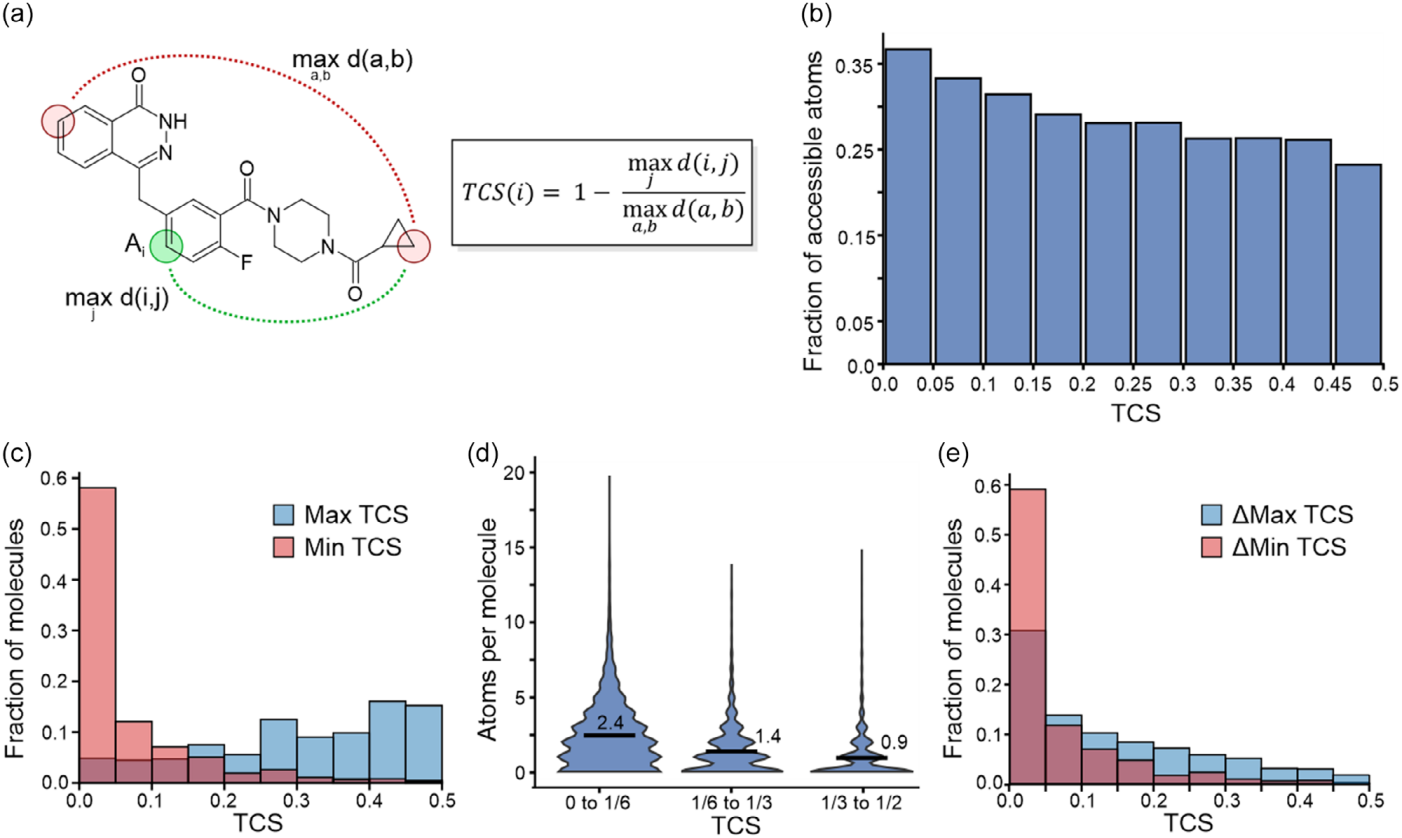
Relationship between the location of a position within a molecule and its accessibility. (a) Definition of the topological center score (TCS). (b) Fraction of accessible positions as a function of TCS. (c) Distribution of maximal (red) and minimal (blue) TCS values across protein–ligand complexes. (d) Violin plots showing the number of accessible positions per molecule for different TCS ranges. (e) Difference between maximal and minimal TCS values for accessible and modifiable positions.

The dependence of the accessibility on the position of a modifiable atom within a molecule was investigated. For DELs, this information is relevant because linear libraries have the DNA attached at the periphery, whereas in branched libraries, the linker positioned closer to the center of the molecule [33]. A topological center score (TCS; Figure 3a) was used to describe the relative position of an atom within a molecule as the ratio of the distance to the farthest atom away from this position (max d(*a*,*b*)) and the largest distance between any pair of atoms in the molecule (max d(*i*,*j*)). Peripheral atoms have TCS values below 0.15, whereas atoms closer to the molecular center have TCS values above 0.33.

The fraction of accessible positions inversely correlates with TCS, indicating that peripheral atoms tend to be more accessible than central ones, although the dependency is modest (Figure 3b). 36% of positions with TCS of <0.05 are classified as accessible, whereas this value drops to 29% for positions with TCS of 0.45–0.5. There are also more modifiable atoms toward the ends of molecules than near the center (Figure S1). The combined effect results in a significantly higher number of positions amenable to linker attachment at the periphery than at the center. More than 50% of all molecules contain an accessible atom with a TCS value ≤0.05, whereas only 17.5% of molecules contain an accessible atom with a TCS value ≥0.45 (Figure 3c). Overall, molecules have 2.4 accessible positions toward the end (TCS < 1/6) but only 0.92 toward the center (TCS > 0.33; Figure 3d). Furthermore, in more than half of the cases, one of the most peripheral positions is accessible (ΔMax TCS = 0), whereas the fraction is only ~0.3 for the most central atoms (ΔMin TCS = 0; Figure 3e). In conclusion, opportunities to modify protein ligands occur more frequently at peripheral positions than near the molecular center, suggesting that linear libraries may be preferable to branched ones from the perspective of linkerability.

### 2.3 Geometry of Solvent Directed Spaces

The above analysis is limited to general solvent accessibility and the availability of space to introduce the first atom of a linker. However, for buried positions there may be no viable path for a linker to reach the attachment site, and the resulting rigidification of the linker may impose a thermodynamic penalty that disfavors binding [34].

**FIGURE 4:**
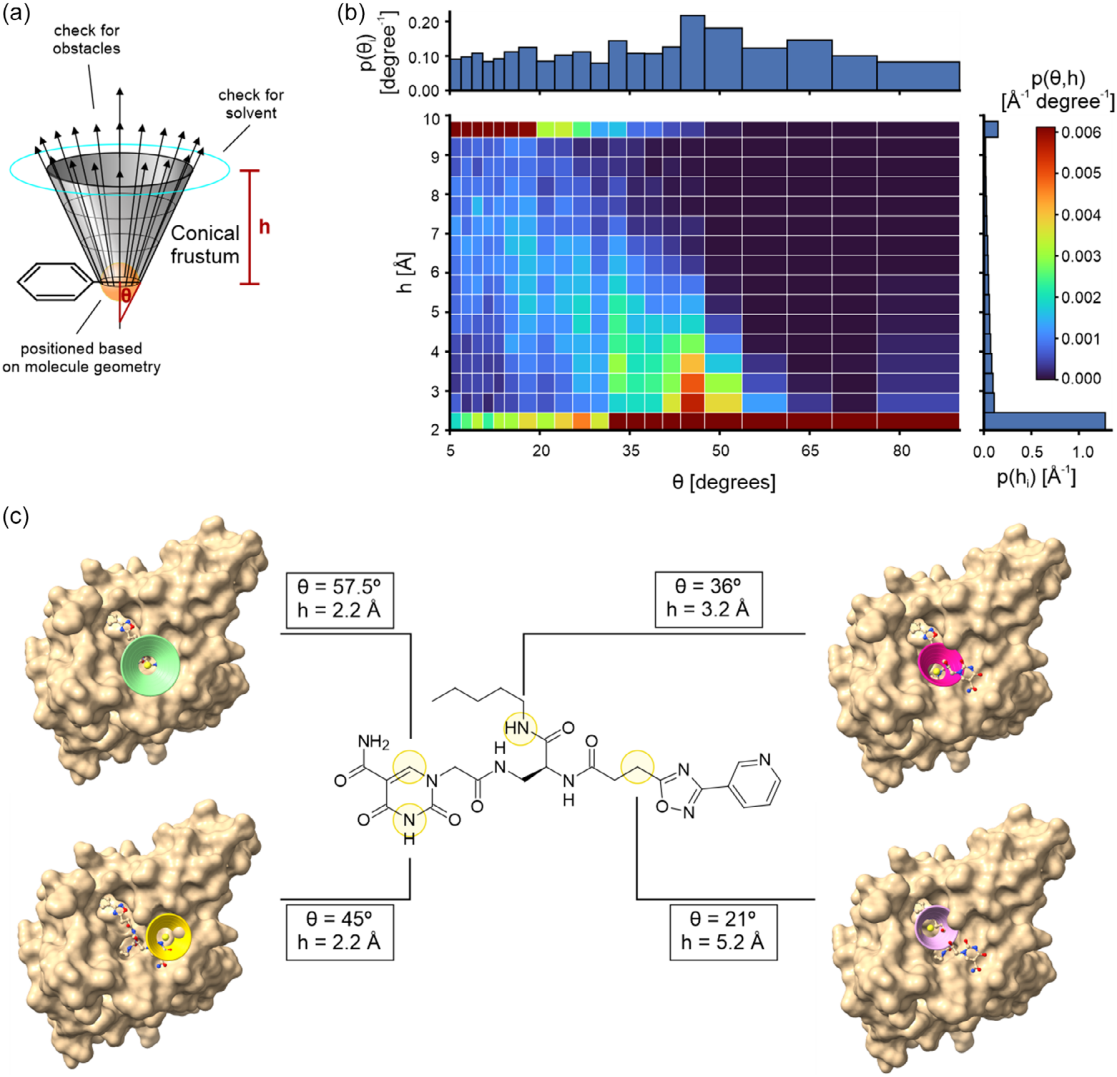
Analysis of the geometry of solvent directed space. (a) To enable comparison across large numbers of sites, conical frustums were used to approximate the local solvent‐directed environment. Cones with a base radius corresponding to the first linker atom and a small surrounding space were fitted to the protein surface to minimize steric clashes, and the cone height was defined as the distance between the linker atom and the protein surface. Each frustum is described by two parameters: the cone angle *θ* and the height *h*. (b) Distribution of *θ* and *h* across 35 227 analyzed binding sites (for 4270 sites no cone could be fitted), shown as a probability density heatmap with marginal histograms. The colormap for the heatmap is truncated at 0.95 to enhance visibility. (c) Example solvent cones identified for a DEL‐derived SIRT6 inhibitor, shown for illustration.

A method was therefore developed for the high‐throughput assessment of solvent pocket geometry around putative linker attachment sites using simple, interpretable parameters. Conceptually, the solvent‐directed space around a potential linker attachment atom corresponds to the region through which a linker could extend without colliding with the protein. To capture this geometry, the accessible space was approximated as a conical frustum extending outward from the attachment atom, described by the cone angle *θ* and height *h* (Figure 4a). The cone angle reflects the width of the exit path from the binding pocket, whereas the cone height reflects how deeply the atom is buried in the protein.

The algorithm estimates the local escape direction from the surrounding protein and ligand geometry and samples candidate cone axes within a spherical cap around this direction to efficiently identify the optimal orientation. The frustum is anchored at the target group with the cone base at the atom center and the tip placed behind the atom along the axis such that the cone radius equals the group's exclusion radius. For each axis and cone angle, the algorithm evaluates whether the truncated frustum is sufficiently open by computing minimum distances between obstacle points and ray segments on the cone surface and interior. The largest cone angle satisfying these constraints is selected. The frustum height is then determined by advancing the cone outward along the axis until the pocket becomes solvent‐exposed (see Supporting Information for details).

The 39 497 positions identified as accessible were analyzed using this algorithm across a grid of predefined values for *θ* and *h*. For 4270 positions (10.8%), no cone satisfying the steric criteria could be identified even at the minimal tested cone angle of 6°, indicating that no continuous sterically allowed path to solvent exists. The resulting probability density distribution of frustum parameters p(*θ*,*h*) is shown as a heatmap in Figure 4b (for the actual distribution of p(*θ*,*h*), see Figure S2), with marginal distributions for height and angle displayed as bar plots. Examples of identified cones are shown for a DEL‐derived SIRT6 inhibitor [35]. Although cones are observed across a wide range of geometries, two main populations emerge. The first consists of cones with the minimal height allowed in the model (2.2 Å), which account for 57% of all cones and almost all the cones with wide angles (*θ* > 45°). These correspond to atoms located close to the protein surface, where the linker exit path reaches solvent almost immediately. The second population forms a diagonal distribution from moderate angles and shallow depths to progressively narrower and deeper cones. These cones correspond to atoms located deeper in binding pockets, where the linker must pass through increasingly narrow channels before reaching solvent.

**FIGURE 5:**
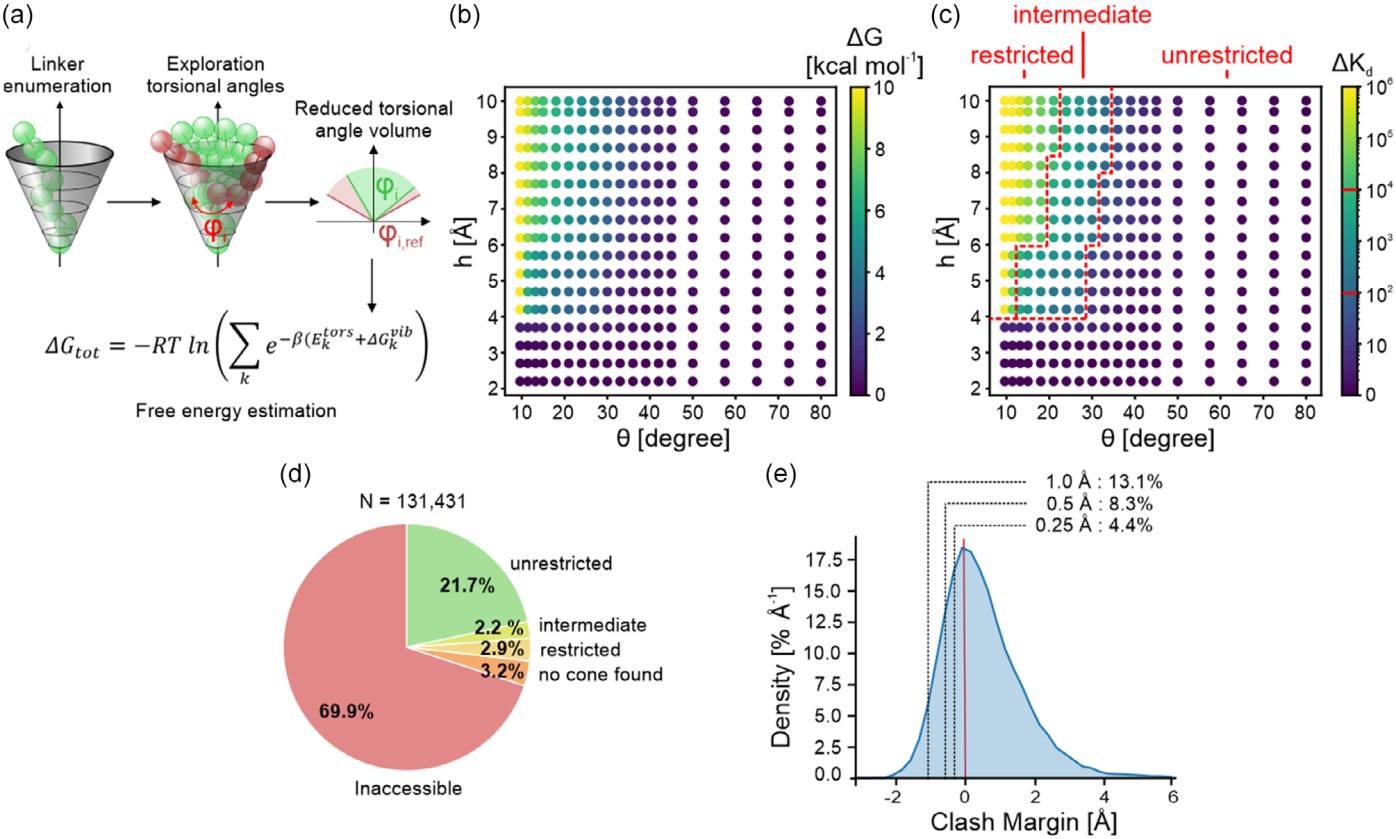
Estimation of the free energy penalty caused by restricting a flexible linker within conical frustums. (a) Geometry‐based statistical mechanics model used to estimate free energy penalties associated with linker confinement (see Supporting Information for details). (b) Free energy penalty for fitting a C8 alkane linker (typical length for molecular chimeras and in DELs) into cones of specific cone angles *θ* and heights *h*. (c) Estimated model‐based loss of binding affinity for a C8 alkane linker as a function of cone geometry. The predicted reduction in binding affinity was used to classify positions as restricted (Δ*K*_d_ > 10^4^), intermediate (10^4^ ≥ Δ*K*_d_ > 10^2^), and unrestricted (Δ*K*_d_ ≤ 10^2^; boundaries specified as dashed red lines). (d) Classification of modifiable positions in protein–ligand complexes based on geometric accessibility and the predicted geometry‐based loss in binding affinity (*N* = number of positions). (e) Density of the clash margin between the probe sphere used to assess local space (Figure 2a) and the protein surface, serving as an indicator of linkerability enabled by local distortions of the protein–ligand complex.

Intuitively, linker positioning becomes increasingly challenging as binding sites become narrower and deeper, but the quantitative impact of this effect is unknown. To facilitate classification of positions and gain physical insight, a geometry‐based statistical‐mechanics model was developed that combines torsional energetics with steric confinement imposed by the pocket to estimate the thermodynamic cost of positioning a flexible alkyl linker within a protein pocket (Figure 5a).

In this model, the linker is represented as a chain of methylene groups with tetrahedral geometry, and its conformational space is sampled by enumerating discrete backbone torsional states. For each conformation, steric compatibility with the pocket is evaluated using a simplified geometric representation in which accessible space is approximated by a conical frustum extending from the attachment point with a flat surface at the mouth. Conformations that violate geometric constraints or have self‐clashes are discarded, while the remaining states are assigned free energies consisting of intrinsic torsional contributions and an additional confinement term reflecting the reduced configurational space. To find the free energy of the linker when its endpoints are fixed in a certain geometry, we consider every possible way the linker could bend or arrange itself, combine their statistical contributions using the partition function, and compute the free energy from that. Comparison with a reference geometry corresponding to an unconstrained cone provides an estimate of the internal free energy penalty associated with linker confinement. Details of the statistical mechanics model are provided in the Supporting Information. The model is not intended to provide quantitative free‐energy estimates, as the approximation of solvent‐directed spaces is an oversimplification and the model neglects important contributions such as enthalpic interactions with the protein and entropic effects associated with solvent displacement. In addition, the analysis treats the protein–ligand complex as rigid, whereas both partners may undergo conformational adjustments upon linker attachment. Lastly, the model requires selecting specific values for key parameters, and these choices strongly influence the predicted energy penalties. Consequently, the absolute free‐energy values are meant to be interpreted only qualitatively. Nevertheless, the model captures the dominant entropic penalty associated with restricting linker conformations within confined geometries and therefore provides physically meaningful trends that allow classification of potential linker attachment sites.

The free energy penalties were calculated across a grid of accessible frustum angle and height combinations (the same grid used in the cone detection algorithm; Figure 5b). Low penalties were observed for large cone angles (>40°) and small heights, consistent with the low entropic cost of putting a linker into large, shallow solvent cones. As the cones become narrower, a steep increase in free energy is observed, corresponding to a significant predicted decrease in binding affinity.

The calculated free energies allowed assignment of all cone geometries obtained (Figure 4b) to a predicted linkerability class. The predicted reduction in binding affinity was used to classify positions as restricted (ΔK_d_ > 10^4^), intermediate (10^4^ ≥ Δ*K*_d_ > 10^2^), and unrestricted (ΔK_d_ ≤ 10^2^; boundaries indicated by dashed red lines in Figure 5c). Based on these criteria, 21.7% of all positions fall into the unrestricted category, 2.2% into the intermediate category, and 2.9% are classified as restricted. Therefore, approximately one in five modifiable atoms is fully accessible for linker attachment without requiring distortion of the protein–ligand complex.

All analyses above treated the protein–ligand complex as rigid with hard surfaces. In reality, both ligands and proteins may undergo conformational changes that allow linker positioning while maintaining binding of the small molecule to the protein, meaning that the present approach underestimates the number of linkerable sites. Generalizable estimation of such effects is difficult, as flexibility depends on the specific interactions between the protein and ligand and therefore requires detailed analysis such as molecular dynamics simulations.

As a first approximation, the distribution of clash margins used in the solvent accessibility algorithm (Figure 2a) can provide insight into the fraction of positions that may become accessible with minimal conformational changes. This assumption is based on the hypothesis that the lower the clash margin, the smaller the structural adjustment required to allow linker introduction. Analysis of the observed values shows that 8.3% of positions have a clash margin <0.5 Å, increasing to 13.1% at <1.0 Å. At least part of these positions are expected to be linkerable (Figure 5e). Most positions classified as inaccessible were defined as such because of low solvent‐accessible surface area rather than limited local spacing, which this analysis does not capture.

### 2.4 Linker Accessibility Across Target Classes

**FIGURE 6:**
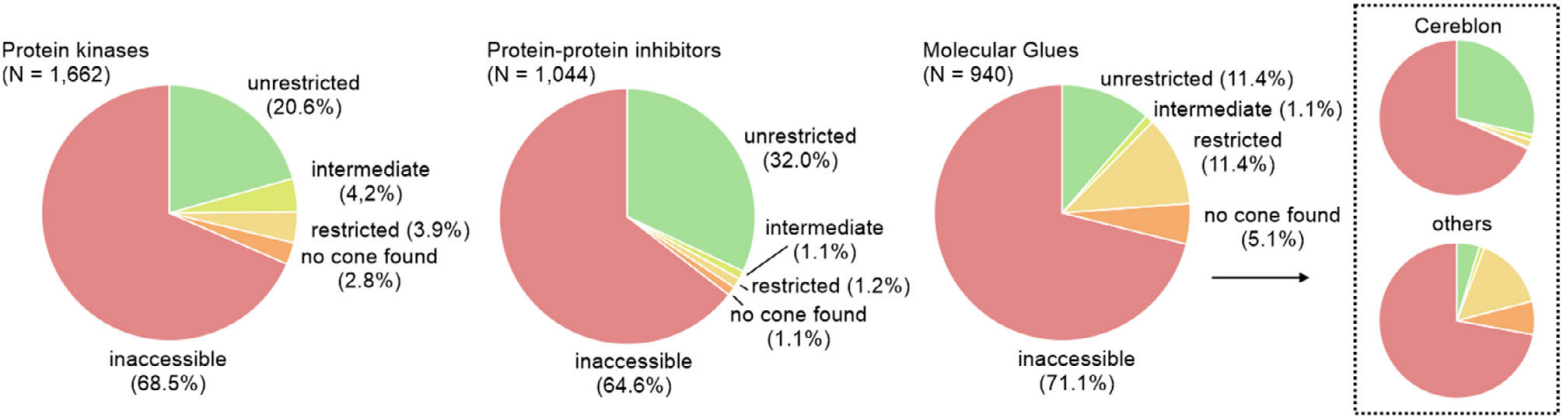
Classification of binding sites regarding linkerability across different target classes, protein kinases, inhibitors of protein–protein interactions, and molecular glues (*N* = number of positions).

The analysis above is based on a random set of protein–ligand complexes from the RCSB Protein Data Bank. To confirm that these values are representative of medicinal chemistry targets and to benchmark linker accessibility across different target classes, the analysis was repeated using structures of protein kinases, inhibitors of protein–protein interactions (PPIs), and molecular glue molecules [36] (Figure 6). The distribution of linker accessibility categories for kinases, a classical medicinal chemistry target, is similar to that of the general population, indicating that the results are representative. For PPIs, the fraction of accessible binding sites is increased, reflecting their typically shallow binding pockets and larger ligands, whereas for molecular glues it is smaller, with many ligands being buried at protein–protein interfaces. This trend becomes clearer when separating Cereblon binders, which are partially surface‐exposed [37], from other molecular glues that tend to exhibit low accessibility. Interestingly, the fraction of inaccessible positions remains relatively constant across the datasets, while the largest variation is observed for unrestricted sites, ranging from 32.0% for PPIs to 20.6% for protein kinases and 4.7% for non‐Cereblon molecular glues. Importantly, molecular glues, in contrast to PROTACs, do not contain an intrinsic linker. Here, linkerability is relevant in the context of DELs, where the required DNA‐attachment linker must be tolerated by both proteins interacting simultaneously with the small molecule.

Together, these results confirm the validity of the global linker accessibility analysis and reveal trends across target classes consistent with the expected differences in binding‐site geometries.

### 2.5 Interpretation of Linker Accessibility in the Context of DEL Data and PROTACs

**FIGURE 7:**
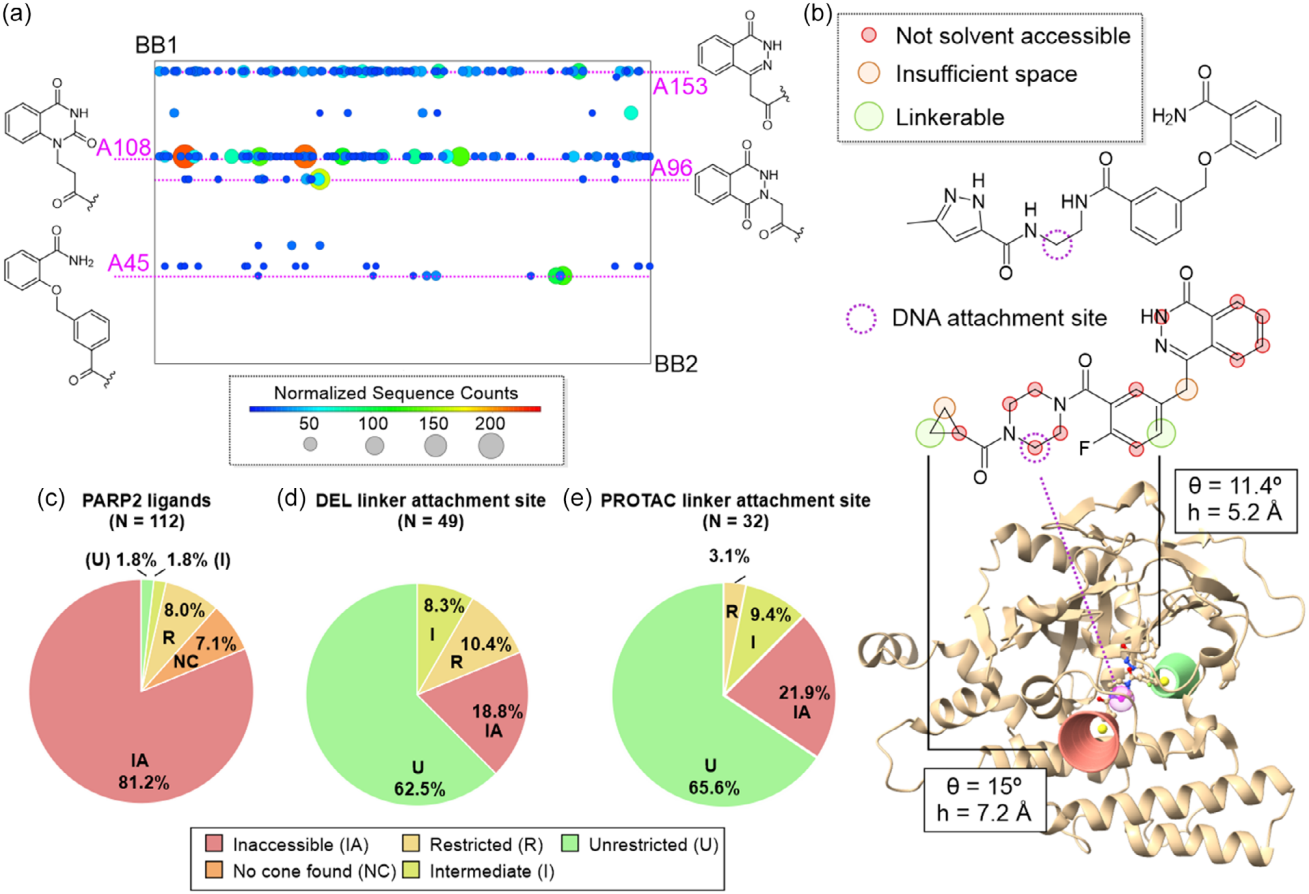
Contextualization of the linkerability analysis in DELs in light of PARP2 data. (a) Enrichment fingerprint of DEL compounds for PARP2, revealing four dominant fragments, with only subsets of the corresponding full structures appearing as hits. For details see Ref. [31]. (b) Structure of a representative hit compound and its relationship to the PARP inhibitor Olaparib. The linkerability status of modifiable positions on Olaparib is indicated, and the identified frustums are shown in the corresponding cocrystal structure (PDB: 4VTJ). (c) Classification of linker accessibility for positions in nine ligands observed in PARP2 cocrystal structures. (d) Classification of linker accessibility for DNA‐attachment position in DEL hit molecules (Table S1). (e) Classification of linker accessibility for the PROTAC linker attachment position of parent protein ligands (Table S2).

Having established the overall analytical framework and obtained a global view of the surface accessibility of protein–ligand costructures, we next sought to place these findings in the context of DEL results. The case of a selection of a focused DEL for PARP2 was considered [31]. Four heterocycles at position 1 dominated the enriched compounds, with number of hits differing substantially across the four, with many for A108 and A153 and only very few for A45 and A96 (Figure 7a). Notably, the inhibitory potency of the compounds was predominantly determined by these four building blocks, with enrichment not having significant predictive power [31]. Furthermore, analysis of nonhits revealed the presence of numerous false negatives. These observations were at least partly attributed to interference of the linker with ligand binding. However, it remained unclear to what extent this example reflects a general phenomenon in DEL selections or represents an extreme case arising from limited linker accessibility.

The cocrystal structure of PARP2 and Olaparib (PDB: 4TVJ) [38] was analyzed with the workflow developed here based on the observation that A45‐containing molecules are structurally closely related to this drug (Figure 7b). Of the 16 modifiable positions, only two were classified as solvent‐accessible. Moreover, these two positions corresponded to deep and narrow cones (*θ* = 11.4° and *h* = 5.2 Å; *θ* = 15° and *h* = 7.2 Å). Structural alignment with Olaparib allows estimating the position of the DNA‐attachment linker of A45‐containing compounds in the PARP2 structure (indicated in pink; Figure 7b). The algorithm predicts this position to be inaccessible. The workflow was then applied to a set of nine cocrystal structures of PARP2 with bound ligands. Overall, PARP2 ligands were found to be significantly less linkerable than those in the overall dataset, with 81.2% of positions classified as inaccessible, 7.1% for which no cones could be identified, and 8.0% associated with restricted cones, leaving only two cones each (1.8%) classified as intermediate and unrestricted (Figure 7c). These results indicate that although PARP2 is a highly druggable protein [39], the introduction of linkers into its ligands is considerably more constrained than in the general population.

To further assess the importance of linkerability in DEL research, we analyzed the accessibility of DNA‐attachment sites in DEL hits. A total of 49 cocrystal structures for which the DNA‐attachment site could be assigned with high confidence were analyzed using the linkerability workflow (Table S1). The overall accessibility distribution of all positions in DEL hits closely resembled that of the general ligand population, with 72.7% classified as inaccessible and 20.2% as unrestricted (Figure S3). In contrast, DNA‐attachment sites showed greatly enhanced linkerability, with only 18.8% being classified as inaccessible and 62.5% as unrestricted. This finding is consistent with the expectation that successful DNA‐attachment sites preferentially point toward solvent. Visual inspection of the nine attachment sites classified as inaccessible revealed that three were solvent‐facing, suggesting flagging by clash margins used by the model (Figure 5e). In the remaining cases, the attachment site was buried and frequently oriented toward the protein, indicating that the binding mode of the DEL‐bound compound may differ from that of the corresponding off‐DNA ligand. Notably, these cases were predominantly associated with attachment sites having high TCS values consistent with the greater accessibility of peripheral atoms (Figure 3), whereas attachment at more central positions is more likely to require structural adaptation. Overall, these results highlight the critical role of linkerability in DEL hit discovery and further support the validity of the proposed models.

The accessibility of linker‐attachment sites in protein ligands used for PROTAC development was assessed in a similar manner (Figure 7e). A set of 32 representative matched pairs consisting of PROTACs and their corresponding parent ligands, for which cocrystal structures were available, was assembled (Table S2). The PROTAC linker‐attachment site was identified on each parent ligand, and its linkerability was evaluated using the workflow described above. Consistent with expectations, the majority of linker‐attachment sites were classified as unrestricted (65.6%), indicating solvent exposure. Nevertheless, 21.9% of attachment sites were classified as inaccessible, with several positions oriented toward the protein surface or involved in stabilizing interactions such as charge–charge contacts. The overall distribution across different linkerability categories of the parental ligands is close to that of the generic structure set (Figure S4). Remarkably, the linkerability profiles of DEL DNA‐attachment sites and PROTAC linker‐attachment sites were comparable (Figure 7d,e). These results indicate that DEL hits not only provide target‐binding ligands but are also intrinsically enriched for compounds possessing suitable linker‐attachment vectors, making them attractive starting points for PROTAC and molecular chimera development.

## 3 Discussion and Conclusions

The present study analyzes linkerability across putative modifiable positions in protein–ligand cocrystal structures. An algorithm was developed to classify these positions based on factors affecting linker access, including solvent‐accessible surface area, space for the introduction of linker attachment sites, and solvent‐accessible space approximated by fitted cones. The intentionally simplified model enables analysis of large numbers of complexes while producing easily interpretable and generalizable conclusions. Importantly, the analysis places previously observed linker‐related effects in DEL experiments into a broader structural context, showing that linker accessibility varies widely across targets and ligand positions rather than representing isolated anomalies.

**FIGURE 8:**
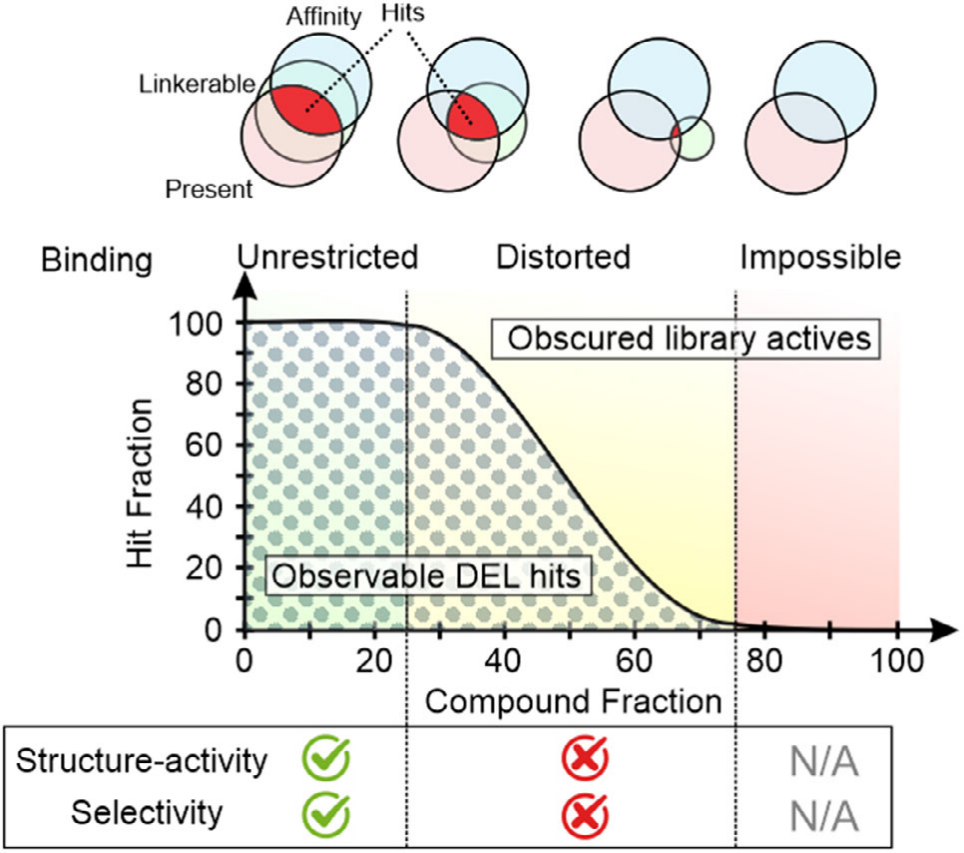
Schematic overview of the impact of linkerability on DEL data. DEL compounds can be divided into three regimes based on linker accessibility. Approximately one quarter of compounds exhibit unrestricted linker accessibility and can, in principle, provide information on structure–activity relationships and target selectivity. Another fraction of compounds may fail to appear as hits despite having intrinsic affinity for the target, because the DNA‐attachment linker is incompatible with the binding interaction. The remaining compounds fall into an intermediate regime, where linker attachment is possible but requires distortion from the optimal binding geometry of the parent ligand. In this regime, only a subset of compounds within clusters of structurally related compounds will be observed as hits, and enrichment is unreliable for extracting structure–activity or selectivity information. The depicted curve represents a qualitative estimate and is expected to depend on the specific library and target.

The results emphasize that linker accessibility acts as a previously underappreciated structural variable influencing DEL outcomes. Despite the model's simplicity, several conclusions can be drawn:

‐ Only a fraction of molecular sites is available for linker attachment. The model estimates that about one fifth of modifiable positions can be modified without restriction, whereas roughly 70% are classified as inaccessible. In the context of DELs, this implies that many DNA‐conjugated molecules with genuine binding affinity may fail to appear as hits and instead manifest as false negatives. The precise values remain uncertain because the model assumes rigid protein–ligand complexes, an assumption that rarely holds. Rather than a strict classification of accessibility, the categories should therefore be interpreted as indicating the degree of structural distortion required to accommodate a linker. Less solvent‐accessible positions likely require greater distortions, and strong binders may better tolerate the associated energetic penalties than weak ones. However, the extent to which such distortions occur in practice, and how they would alter the predicted accessibility distribution, cannot be determined from the present analysis.
‐ A key question in the DEL field is which library design principles enable discovery success. The analysis indicates that linkers attached at peripheral positions are more likely to be tolerated than central linkers. This suggests that linear libraries may be favored over branched libraries, consistent with the distribution of observed DEL hits [33]. Nevertheless, linker positioning represents only one aspect of library design. Reaction chemistry, building block diversity, and complementarity with the target must also be considered.

‐ DEL selections are increasingly used as an indicator of target druggability, with enrichment of library members (sometimes referred to as DELability) often interpreted as evidence that a target is chemically tractable. The present results suggest that differences in linker accessibility distort the relationship between DELability and chemical tractability. Targets whose ligands provide few geometrically favorable linker attachment sites may yield weaker or more variable DEL enrichment even when high‐affinity ligands exist. Consequently, DEL performance may reflect not only target druggability but also the linkerability of the corresponding binding sites.
‐ The data also contextualize the previously observed high rate of false negatives in DEL results for the PARP2 target [31]. The structural analysis presented here indicates that PARP2 lies toward the low‐linkerability end of the spectrum of protein targets. In such environments, linker placement is frequently constrained by the protein surface, making distortions of the binding geometry necessary for many encoded molecules. The previously observed DEL behavior can therefore be understood as an example of how severe linker inaccessibility can shape DEL outcomes.
‐ The discovery of A45‐containing hits for PARP2 despite an unfavorable linker position demonstrates that molecules with linkers at positions classified as inaccessible can still produce DEL hits. Consequently, the present analysis may underestimate the true frequency of accessible positions in protein binders.
‐ ML‐approaches are increasingly used to predict lead compounds from DEL data [14, 29]. These methods implicitly assume that DEL datasets contain structure–activity relationships that can be learned. The present analysis suggests that the reliability of such patterns depends strongly on linker accessibility (Figure 8). When linkers are attached at positions with limited accessibility, observed hit/no‐hit patterns may primarily reflect the ability of different structures to distort and accommodate the linker rather than intrinsic binding affinity. In such cases, the resulting correlations represent structure‐linkerability relationships rather than true structure–activity relationships. Conversely, when linkers are attached at unrestricted sites, DEL data are more likely to reflect genuine affinity trends and thus provide a more suitable basis for machine‐learning models.
‐ Relatedly, DELs are also used to provide information on target selectivity [40]. Based on the insights from the present studies, such efforts can only provide valid information if the linker attachment site is solvent‐exposed, whereas any information for restricted or buried sites would be unreliable.
‐ The DEL discovery process simultaneously selects for target binding and linkerability, as indicated by the high fraction of unrestricted attachment sites (Figure 7d). The comparable distribution of linkerability categories between DEL hits and PROTACs illustrates the general utility of DEL hits to be used for the development of molecular chimeras.

Taken together, the data provide a comprehensive view of DEL molecules (Figure 8). Approximately one‐quarter of molecules can accommodate a solvent‐exposed linker without significant constraints. In these cases, groups of closely related molecules with unrestricted linkerability can reveal meaningful structure–activity and selectivity relationships, assuming the library is homogeneous and the selection process captures these patterns. Another quarter of molecules may fail to appear as hits even when they have high target affinity, simply because the linker interferes with binding. The remaining ~50% of molecules fall into an intermediate linkerability category. For some, binding is still possible because the protein–ligand complex can distort to accommodate the linker; for others, this is not feasible. As linker accessibility decreases, the likelihood of binding despite an unfavorable linker correspondingly declines. Within this intermediate group, structure–activity or selectivity patterns are generally not reliable.

Having identified linkerability as a factor that distorts the relationship between sequence enrichment and actual binding affinity, an important question is how this problem can be overcome when analyzing DEL data. In practice, structural information is unavailable for DEL compounds, and the present geometric analysis is therefore not applicable to predict linker effects for individual molecules. Instead, the results indicate that DEL datasets are likely to contain mixtures of reliable and unreliable structure–activity information arising from differences in linker accessibility. DELs with different attachment linkers have been proposed, but there is no evidence that such libraries provide a benefit. Alternatively, a key to enhancing DEL‐ML based predictions may be to estimate the reliability of specific subdata, allowing for making appropriate corrections, for example, as part of data balancing steps [31]. Given the large size and diversity of DEL datasets, identifying such patterns at the level of data subsets may be more tractable than predicting linker effects for individual compounds. In particular, we envision that cross‐dataset learning approaches could be used to identify and partially correct low‐reliability regions in DEL datasets, and efforts in this direction are ongoing.

Beyond interpreting DEL data, the present analysis provides a general framework for prioritizing linker attachment sites in small‐molecule ligands. These findings are consistent with observations in heterobifunctional molecule design, where linker attachment at solvent‐exposed positions is critical for maintaining target engagement and where linker flexibility and geometry strongly influence activity. Selecting appropriate exit vectors is a central challenge in the design of bifunctional molecules such as PROTACs, and small‐molecule conjugates, where attachment at unsuitable positions can disrupt binding to the target protein. The linkerability metrics introduced here offer a simple structural filter for identifying ligand positions most likely to tolerate linker attachment without major distortion of the binding mode. This approach, therefore, provides a computationally inexpensive strategy for guiding linker placement prior to synthetic exploration.

## Conflicts of Interest

Leash Bio ‐ ScientificAdvisor Structure Therapeutics ‐ Scientific Advisor Philogen SpA ‐ Shareholder Genetech ‐ Speaker fee.

## Supporting information

The source code used in this study is freely available through GitHub at: https://github.com/SnowyTheWestie/LinkerabilityAnalysis.

Supporting Data and Code

## Data Availability

The data that support the findings of this study are openly available in SnowyTheWestie/Linkerability at https://github.com/.

## References

[1] Y. Duan , M. Y. Cai , J. Xu , and Q. Hu , “Rational Design of the Linkers in Targeting Chimeras,” Chemical Science 16 (2025): 17595–17610. 40988686 10.1039/d5sc04859a PMC12451458

[2] E. A. King , M. Meyers , and D. K. Nomura , “Induced Proximity‐Based Therapeutic Modalities,” Nature Reviews Drug Discovery 25 (2026): 175–203. 41174297 10.1038/s41573-025-01316-z PMC13170557

[3] M. Bekes , D. R. Langley , and C. M. Crews , “PROTAC Targeted Protein Degraders: the past Is Prologue,” Nature Reviews Drug Discovery 21 (2022): 181–200. 35042991 10.1038/s41573-021-00371-6 PMC8765495

[4] M. G. Costales , B. Suresh , K. Vishnu , and M. D. Disney , “Targeted Degradation of a Hypoxia‐Associated Non‐Coding RNA Enhances the Selectivity of a Small Molecule Interacting with RNA,” Cell Chemical Biology 26 (2019): 1180–1186.e5. 31130520 10.1016/j.chembiol.2019.04.008 PMC6697612

[5] N. J. Henning , L. Boike , J. N. Spradlin , et al., “Deubiquitinase‐Targeting Chimeras for Targeted Protein Stabilization,” Nature Chemical Biology 18 (2022): 412–421. 35210618 10.1038/s41589-022-00971-2 PMC10125259

[6] S. U. Siriwardena , D. N. P. M. Godage , V. M. Shoba , et al., “Phosphorylation‐Inducing Chimeric Small Molecules,” Journal of the American Chemical Society 142 (2020): 14052–14057. 32787262 10.1021/jacs.0c05537

[7] W. J. Gibson , A. Sadagopan , V. M. Shoba , A. Choudhary , M. Meyerson , and S. L. Schreiber , “Bifunctional Small Molecules That Induce Nuclear Localization and Targeted Transcriptional Regulation,” Journal of the American Chemical Society 145 (2023): 26028–26037. 37992275 10.1021/jacs.3c06179 PMC10704550

[8] C. E. Jakobsche , C. G. Parker , R. N. Tao , M. D. Kolesnikova , E. F. Douglass Jr. , and D. A. Spiegel , “Exploring Binding and Effector Functions of Natural Human Antibodies Using Synthetic Immunomodulators,” ACS Chemical Biology 8 (2013): 2404–2411. 24053626 10.1021/cb4004942 PMC3830660

[9] S. Cazzamalli , E. Puca , and D. Neri , “Past, Present and Future of Drug Conjugates for Cancer Therapy,” Nature Cancer 6 (2025): 1494–1504. 40935909 10.1038/s43018-025-01042-w

[10] R. M. Franzini and C. Randolph , “Chemical Space of DNA‐Encoded Libraries,” Journal of Medicinal Chemistry 59 (2016): 6629–6644. 26914744 10.1021/acs.jmedchem.5b01874

[11] A. Gironda‐Martinez , E. J. Donckele , F. Samain , and D. Neri , “DNA‐Encoded Chemical Libraries: A Comprehensive Review with Succesful Stories and Future Challenges,” ACS Pharmacology and Translational Science 4 (2021): 1265–1279. 34423264 10.1021/acsptsci.1c00118 PMC8369695

[12] A. A. Peterson and D. R. Liu , “Small‐Molecule Discovery through DNA‐Encoded Libraries,” Nature Reviews Drug Discovery 22 (2023): 699–722. 37328653 10.1038/s41573-023-00713-6 PMC10924799

[13] C. M. Gampe , B. Worsdorfer , G. Zou , and A. Ricci , “Analyses of Recent Hit‐Finding Campaigns for Difficult Targets Provides Guidance for Informed Integrated Hit Discovery,” ACS Medicinal Chemistry Letters 17 (2026): 484–489. 41704384 10.1021/acsmedchemlett.5c00676 PMC12907905

[14] V. Poongavanam , S. P. Turunen , K. Sandberg , U. Yngve , and J. Wannberg , “Toward Generalizable Predictive Models for DNA‐Encoded Libraries,” Drug Discovery Today 31 (2026): 104629. 41722895 10.1016/j.drudis.2026.104629

[15] K. McCloskey , E. A. Sigel , S. Kearnes , et al., “Machine Learning on DNA‐Encoded Libraries: A New Paradigm for Hit Finding,” Journal of Medicinal Chemistry 63 (2020): 8857–8866. 32525674 10.1021/acs.jmedchem.0c00452

[16] P. Binder , M. Lawler , L. Grady , et al., “Partial Product Aware Machine Learning on DNA‐Encoded Libraries,”ChemArxiv (2022).

[17] K. S. Lim , A. G. Reidenbach , B. K. Hua , et al., “Machine Learning on DNA‐Encoded Library Count Data Using an Uncertainty‐Aware Probabilistic Loss Function,” Journal of Chemical Information and Modeling 62 (2022): 2316–2331. 35535861 10.1021/acs.jcim.2c00041 PMC10830332

[18] F. Xiong , M. Yu , H. Xu , et al., “Discovery of TIGIT Inhibitors Based on DEL and Machine Learning,” Frontiers in Chemistry 10 (2022): 982539. 35958238 10.3389/fchem.2022.982539 PMC9360614

[19] S. Ahmad , J. Xu , J. A. Feng , et al., “Discovery of a First‐in‐Class Small‐Molecule Ligand for WDR91 Using DNA‐Encoded Chemical Library Selection Followed by Machine Learning,” Journal of Medicinal Chemistry 66 (2023): 16051–16061. 37996079 10.1021/acs.jmedchem.3c01471

[20] R. Hou , C. Xie , Y. Gui , G. Li , and X. Li , “Machine‐Learning‐Based Data Analysis Method for Cell‐Based Selection of DNA‐Encoded Libraries,” ACS Omega 8 (2023): 19057–19071. 37273617 10.1021/acsomega.3c02152 PMC10233830

[21] A. L. Montoya , M. Glavatskikh , B. J. Halverson , et al., “Combining Pharmacophore Models Derived from DNA‐Encoded Chemical Libraries with Structure‐Based Exploration to Predict Tankyrase 1 Inhibitors,” European Journal of Medicinal Chemistry 246 (2023): 114980. 36495630 10.1016/j.ejmech.2022.114980 PMC9805525

[22] W. Torng , I. Biancofiore , S. Oehler , et al., “Deep Learning Approach for the Discovery of Tumor‐Targeting Small Organic Ligands from DNA‐Encoded Chemical Libraries,” ACS Omega 8 (2023): 25090–25100. 37483198 10.1021/acsomega.3c01775 PMC10357458

[23] K. Shmilovich , B. Chen , T. Karaletsos , and M. M. Sultan , “DEL‐Dock: Molecular Docking‐Enabled Modeling of DNA‐Encoded Libraries,” Journal of Chemical Information and Modeling 63 (2023): 2719–2727. 37079427 10.1021/acs.jcim.2c01608

[24] C. Zhang , M. Pitman , A. Dixit , et al., “Building Block‐Based Binding Predictions for DNA‐Encoded Libraries,” Journal of Chemical Information and Modeling 63 (2023): 5120–5132. 37578123 10.1021/acs.jcim.3c00588 PMC10466377

[25] Y. Suo , X. Qian , Z. Xiong , et al., “Enhancing the Predictive Power of Machine Learning Models through a Chemical Space Complementary DEL Screening Strategy,” Journal of Medicinal Chemistry 67 (2024): 18969–18980. 39441849 10.1021/acs.jmedchem.4c01416

[26] S. Han , X. Guo , M. Wang , et al., “Highly Selective Novel Heme Oxygenase‐1 Hits Found by DNA‐Encoded Library Machine Learning beyond the DEL Chemical Space,” ACS Medicinal Chemistry Letters 15 (2024): 1456–1466. 39291011 10.1021/acsmedchemlett.4c00121 PMC11403747

[27] B. Chen , M. M. Sultan , and T. Karaletsos , “Compositional Deep Probabilistic Models of DNA‐Encoded Libraries,” Journal of Chemical Information and Modeling 64 (2024): 1123–1133. 38335055 10.1021/acs.jcim.3c01699

[28] J. Wellnitz , S. Ahmad , N. Bagale , et al., “Enabling Open Machine Learning of Deoxyribonucleic Acid‐Encoded Library Selections to Accelerate the Discovery of Small Molecule Protein Binders,” Journal of Medicinal Chemistry 68 (2025): 21635–21648. 41047897 10.1021/acs.jmedchem.5c01972 PMC12557371

[29] M. Wichert , L. Guasch , and R. M. Franzini , “Challenges and Prospects of DNA‐Encoded Library Data Interpretation,” Chemical Reviews 124 (2024): 12551–12572. 39508428 10.1021/acs.chemrev.4c00284

[30] A. L. Satz , “Simulated Screens of DNA Encoded Libraries: The Potential Influence of Chemical Synthesis Fidelity on Interpretation of Structure–Activity Relationships,” ACS Combinatorial Science 18 (2016): 415–424. 27116029 10.1021/acscombsci.6b00001

[31] A. L. Montoya , A. S. Hogendorf , S. Tingey , et al., “Widespread False Negatives in DNA‐Encoded Library Data: How Linker Effects Impair Machine Learning‐Based Lead Prediction,” Chemical Science 16 (2025): 10918–10927. 40395382 10.1039/d5sc00844a PMC12086585

[32] S. Mitternacht , “FreeSASA: An Open Source C Library for Solvent Accessible Surface Area Calculations,” F1000Research 5 (2016): 189. 26973785 10.12688/f1000research.7931.1 PMC4776673

[33] W. K. Weigel , A. L. Montoya , and R. M. Franzini , “Evaluation of the Topology Space of DNA‐Encoded Libraries,” Journal of Chemical Information and Modeling 63 (2023): 4641–4653. 37493573 10.1021/acs.jcim.3c01008 PMC11092675

[34] C. E. Chang , W. Chen , and M. K. Gilson , “Ligand Configurational Entropy and Protein Binding,” Proceedings of the National Academy of Sciences 104 (2007): 1534–1539. 10.1073/pnas.0610494104 PMC1780070 17242351

[35] W. You , A. L. Montoya , S. Dana , R. M. Franzini , and C. Steegborn , “Elucidating the Unconventional Binding Mode of a DNA‐Encoded Library Hit Provides a Blueprint for Sirtuin 6 Inhibitor Development,” ChemMedChem 19 (2024): e202400273. 38940296 10.1002/cmdc.202400273 PMC11486586

[36] X. Wang , Z. Zhuang , C. Zhang , et al., “MolGlueDB: an Online Database of Molecular Glues,” Nucleic Acids Research 54 (2026): D1510–D1518. 40842115 10.1093/nar/gkaf811 PMC12807675

[37] G. Petzold , P. Gainza , S. Annunziato , et al., “Mining the CRBN Target Space Redefines Rules for Molecular Glue–induced Neosubstrate Recognition,” Science 389 (2025): 6736. 10.1126/science.adt6736 40608931

[38] A. G. Thorsell , T. Ekblad , T. Karlberg , et al., “Structural Basis for Potency and Promiscuity in Poly(ADP‐Ribose) Polymerase (PARP) and Tankyrase Inhibitors,” Journal of Medicinal Chemistry 60 (2017): 1262–1271. 28001384 10.1021/acs.jmedchem.6b00990 PMC5934274

[39] X. Lin , W. Jiang , J. Rudolph , B. J. Lee , K. Luger , and S. Zha , “PARP Inhibitors Trap PARP2 and Alter the Mode of Recruitment of PARP2 at DNA Damage Sites,” Nucleic Acids Research 50 (2022): 3958–3973. 35349716 10.1093/nar/gkac188 PMC9023293

[40] R. M. Franzini , A. Nauer , J. Scheuermann , and D. Neri , “Interrogating Target‐Specificity by Parallel Screening of a DNA‐Encoded Chemical Library against Closely Related Proteins,” Chemical Communications 51 (2015): 8014–8016. 25786089 10.1039/c5cc01230a

