# Supplementary material for: Linkerability of Protein Ligands: Insights From Cocrystal Structures and Implications for DNA‐Encoded Libraries
Source: Mol Inform. 2026 Aug 2;45(8):e70045. doi: 10.1002/minf.70045 (PMC13430132; doi:10.1002/minf.70045)
Supplement: Supplementary file 1 — The source code used in this study is freely available through GitHub at: https://github.com/SnowyTheWestie/LinkerabilityAnalysis. [file MINF-45-e70045-s002.pdf]

Supporting Information

Linkerability of Protein Ligands: Insights from Co-Crystal Structures and Implications for DNA-Encoded Libraries

Raphael M. Franzini*

Department of Medicinal Chemistry, College of Pharmacy, 30 S 2000 E, Salt Lake City, UT 84112, USA; raphael.franzini@utah.edu

Table of Contents

**Methodology**	**3**
**Supporting Figures**	**4**
**Linkerability Analysis Workflow**	**6**
**Geometric Free-Energy Model**	**11**
**Supporting Tables**	**19**
**References**	**21**

Methodology

Hardware

Calculations were performed on a standard laptop PC equipped with an Intel Core Ultra 7 155 H processor and 16 GB or RAM.

Artificial Intelligence

ChatGPT 5.3 was used in this study to assist with writing Python code, developing the statistical mechanics model, and implementing the cone-fitting algorithm. All code and mathematical results were verified by the author. ChatGPT was also used for typographic and stylistic revisions of the manuscript and the methodology sections in the Supporting Information. The author assumes responsibility for the accuracy and integrity of all text, data, and code.

Analysis of Linkerability of DEL-Hit Compounds

Co-crystal structures of DNA-encoded library (DEL) hits bound to their target proteins were identified through a literature search. To maximize relevance to the original screening results, the analysis was restricted to structures containing compounds closely related to the reported DEL hits, whereas substantially optimized lead compounds were excluded. Linker-attachment sites were assigned manually based on information reported in the original publications, including the structure of the DEL hit and the position used for DNA attachment. Only compounds for which the linker-attachment site could be assigned with high confidence were included in the final analysis.

Analysis of Linkerability of Ligands Used for PROTAC Development

Matched pairs of PROTACs and their corresponding parent ligands were identified using PROTAC-DB,[1] and a literature search. Candidate compounds were selected to represent a diverse range of target proteins without bias toward specific ligand chemotypes. Inclusion in the analysis required the availability of a co-crystal structure of the parent ligand bound to its target protein and an unambiguously defined PROTAC linker-attachment site. Cases in which the parent ligand structure or linker-attachment site could not be assigned with high confidence were excluded.

Supporting Figures

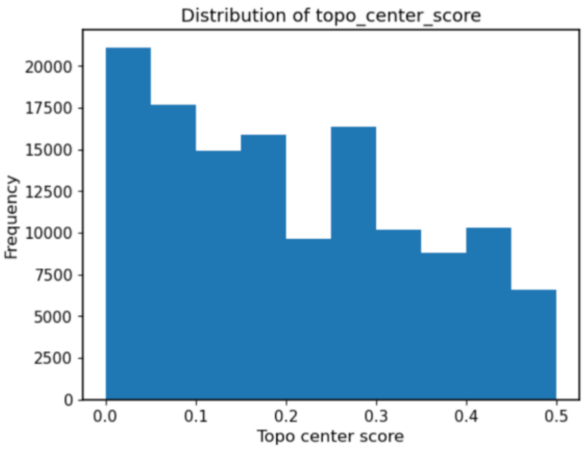

Figure S1. Distribution of the frequency of atoms with as function of topological center score (TCS).

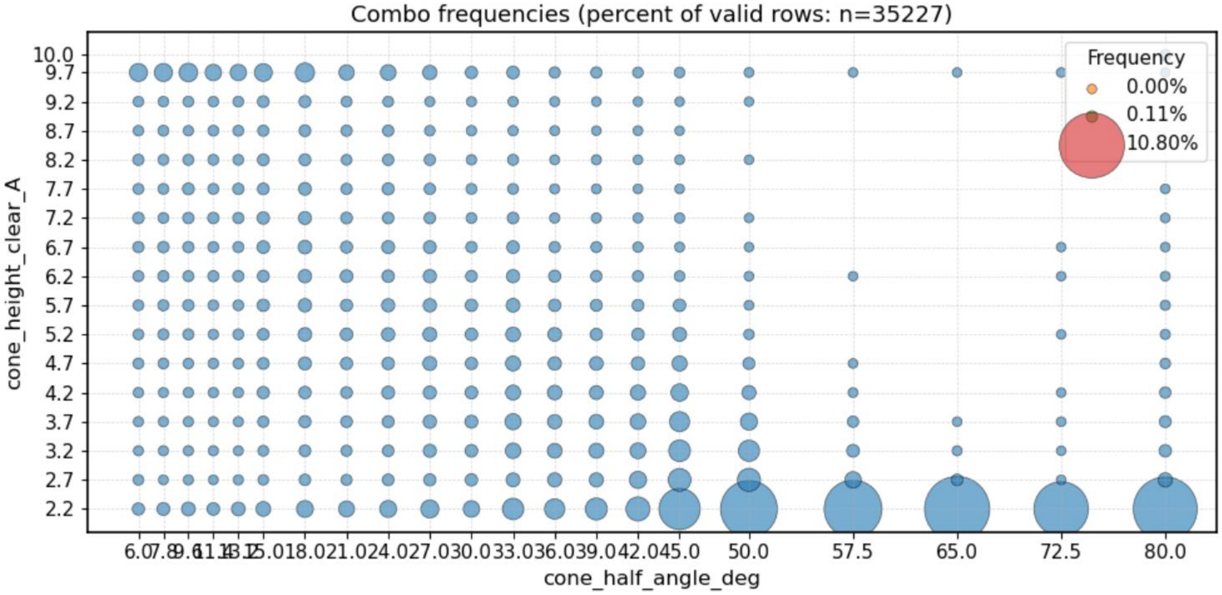

Figure S2. Frequency of cone-angle/height combinations (p(θ,h)) of modifiable positions in protein ligands analyzed in this study.

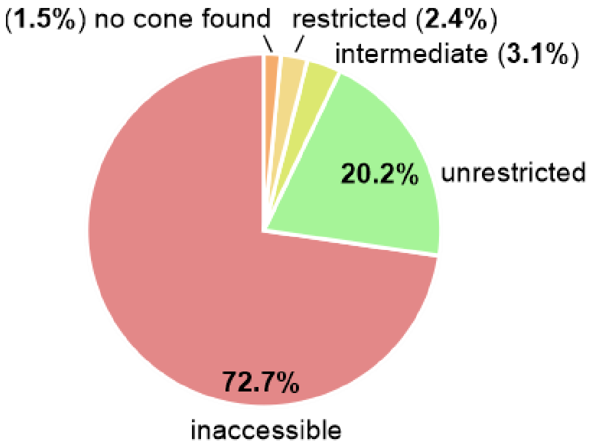

Figure S3. Classification of binding sites regarding linkerability across 49 DEL hits (Table S1; number of positions = 905).

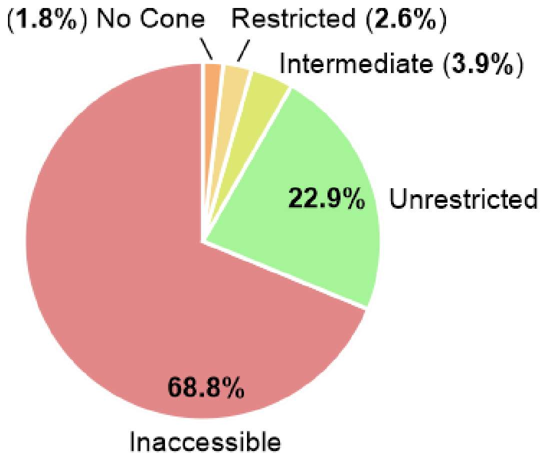

Figure S4. Classification of binding sites regarding linkerability across 32 PROTACs (Table S2; number of positions = 545).

Linkerability Analysis Workflow

Selection of protein-ligand complexes

Protein–ligand complex structures were analyzed from deposited mmCIF files obtained from the RCSB PDB. For each structure, a single bound small-molecule ligand was selected automatically. Candidate residues were first identified from the parsed structure and filtered to exclude waters, common ions, solvent molecules, cofactors, sugars, amino acids, and other non-relevant small components using residue-name exclusion lists. Remaining candidates were further filtered using the RCSB chemical component definition to remove peptide-like species and to retain only ligands within a specified molecular-weight window. If multiple candidates remained, the ligand instance with the largest number of heavy atoms in the deposited structure was selected.

Identification of Modifiable Ligand Positions Prior to Cone Fitting

For the chosen ligand, a chemically complete RDKit representation was reconstructed from the corresponding PDB Chemical Component Dictionary (CCD) definition. Atom identities and bond orders were taken from the CCD record, while atomic coordinates were assigned from the crystallographic residue instance. Explicit hydrogens were then added in RDKit. This yielded a ligand representation with consistent bonding and atom typing while preserving the experimentally observed heavy-atom geometry.

Potentially modifiable positions were identified directly from the hydrogenated ligand graph. Heavy atoms carrying at least one attached hydrogen were classified as candidate attachment atoms, since these positions are in principle accessible by substitution without requiring cleavage of the ligand scaffold.

Solvent exposure

To characterize solvent exposure of these candidate positions, solvent-accessible surface area (SASA) values were computed in the context of the protein complex using FreeSASA. Prior to SASA calculation, problematic metal ions and similar small species were removed from the structure to avoid radius-assignment issues. A custom element-based classifier was used so that both standard protein atoms and ligand atoms from arbitrary chemical component identifiers could be handled consistently. Per-atom SASA values were computed for the full complex, and ligand atoms belonging to the selected residue instance were extracted by chain, residue name, and residue number. These SASA values were then mapped onto the candidate attachment atoms and their associated hydrogen sites.

A preliminary exposure summary was generated from these mapped SASA values. Candidate atoms with SASA above a small threshold were considered exposed at the surface level, while atoms with negligible SASA were treated as buried and were not prioritized for further geometric analysis.

Geometric space analysis for attachment of first atom of linker

Each candidate position was subjected to a local geometric growability test. For each candidate attachment atom, idealized substituent directions were generated from the local ligand geometry using atom hybridization and connectivity. Tetrahedral directions were used for nominally sp3 centers, trigonal planar directions for sp2 and aromatic centers, and linear directions for sp centers. These directions were derived from the heavy-atom neighborhood and did not rely on the deposited hydrogen coordinates. Along each candidate direction, a first introduced atom was placed at a fixed bond-length offset from the attachment atom. The distance from this trial position to the nearest protein heavy atom was then evaluated. A position was considered locally growable if at least one trial direction satisfied the required minimum clearance threshold (3.2 Å).

Topological center score

A ligand-intrinsic topological descriptor (topological center score, TCS) was assigned to each candidate position to distinguish scaffold termini from more central sites. This descriptor was calculated on the heavy-atom molecular graph using the normalized graph eccentricity of each attachment atom. Higher values correspond to more central positions in the ligand graph, whereas lower values indicate more terminal positions.

Identification of Surface-Accessible Conical Volumes

Purpose

To quantify the steric accessibility around selected atoms, a computational procedure was developed to identify the largest surface-exposed conical frustums that can be placed around a given atom without intersecting the surrounding molecular structure. All atoms of the protein–ligand complex were treated as point obstacles with an effective exclusion radius (1.5 Å) used for collision detection.

Cone geometry and positioning

For each atom of interest, the center of the atom defines the base of the cone. The cone apex is placed along the opposite direction of the cone axis such that the cone radius at the atom equals a predefined exclusion radius r_{excl} (2.2 Å), which approximates the steric radius of the atom or functional group. This construction ensures that the cone tangentially touches the atom at its base. To prevent artifacts arising from the immediate neighborhood of the atom, obstacles located within r_{excl} + r_{probe} of the atom are ignored. In addition, obstruction checks begin slightly outside the atom surface so that the internal volume of the atom does not influence the calculation.

Determination of candidate cone orientations

To efficiently identify directions pointing toward solvent-accessible space, a local "escape direction" is estimated from the spatial distribution of nearby atoms. The coordinates of atoms within a local neighborhood are used to compute a covariance matrix of vectors relative to the target atom. The eigenvector corresponding to the smallest eigenvalue provides an estimate of the direction with the lowest surrounding atomic density. Candidate cone axes are then sampled within a spherical cap around this escape direction using points generated on a Fibonacci sphere. This strategy restricts the search to directions that are most likely to lead toward solvent exposure while maintaining an approximately uniform angular sampling.

Identification of the optimal cone angle

For each candidate axis, a series of cone half-angles is evaluated. The cone that satisfies predefined openness criteria with the largest half-angle is selected. To determine whether a cone is sterically accessible, rays are generated along the cone surface and within the interior of the cone (length of rays = 7.5 Å; number of surface rays = 72; number of interior rays = 16). Instead of discrete ray-marching, the algorithm computes the minimum distance between each obstacle and the continuous ray segment defining the cone surface or interior direction. A ray is considered blocked if this minimum distance is smaller than the probe radius r_{probe} (1.5 Å). The fraction of unblocked rays (90%) is used to estimate the openness of the cone. A variable openness threshold is applied that increases with cone angle, allowing narrow cones to tolerate slightly more obstruction while enforcing stricter criteria for wider cones. A coarse sampling stage is first used to rapidly eliminate unsuitable orientations, followed by a full sampling stage for cones that pass the coarse test.

Determination of cone height

Once the optimal cone orientation and angle have been identified, the height of the truncated cone is determined. Starting from the atom surface, the cone is extended outward along its axis in small increments. At each step, points located on a circular ring slightly outside the cone surface (d = 1.75 Å) are evaluated to determine whether the region is exposed to solvent. The distance between these points and nearby atoms is calculated, and the fraction of points that remain unobstructed is determined. The height of the cone is defined as the first distance at which the outer solvent shell becomes sufficiently open (90%), indicating that the cone has emerged into solvent-accessible space.

Computational considerations

To improve efficiency, spatial queries are accelerated using a k-d tree data structure when available. Additionally, obstacle checks are restricted to atoms that could potentially intersect the truncated cone, reducing the number of distance calculations required. The combination of

directed axis sampling, continuous distance evaluation, and spatial filtering enables the rapid identification of accessible conical volumes even for large biomolecular systems.

Geometric Free-Energy Model

Purpose

The purpose of this model is to estimate the free-energy penalty associated with geometric confinement of flexible linkers within restricted regions of protein binding sites. The approach is designed to reveal trends across different pocket geometries and to enable rapid, computationally inexpensive evaluation of multiple potential attachment positions on protein-bound ligands.

Concept

The model consists of the following conceptual steps:

1. The solvent-directed space adjacent to a putative linker attachment site is represented as a conical frustum with rigid boundaries.
2. Linker conformations are enumerated within this conical frustum. Conformations that either intersect the frustum boundaries or exhibit steric self-clashes are eliminated.
3. The free energy of each remaining linker conformation is calculated based on torsional-angle interactions. High-energy conformations are excluded from further analysis.
4. For each retained linker conformation, the range of torsional angles compatible with the geometric constraints is evaluated around the reference torsional state.
5. The accessible torsional ranges across all degrees of freedom are combined to define a multidimensional configuration space describing the local conformational freedom of the linker. Comparison of this accessible configuration space with a reference state without geometric constraints provides an estimate of the confinement-induced loss of conformational entropy.
6. The conformational entropy loss and the torsional free energies are combined using a statistical-mechanical model to obtain the overall free-energy penalty associated with linker confinement.

Geometric representation of the solvent-directed space

The solvent-directed space adjacent to potential linker attachment sites was approximated as a conical frustum with rigid boundaries. The frustum is described by two parameters: the cone half-angle θ and the frustum height h.

A Cartesian coordinate system was defined with the z-axis aligned with the cone axis. The lower plane of the frustum is located at z_0, which corresponds to the position of the linker anchor. The positive direction of the z-axis points from the anchor toward the solvent-exposed mouth of the frustum.

The lower opening of the frustum was chosen such that a sphere representing the first linker bead fits inside the cone with a small margin,

$$r_{\text{frustum}} = 2.2\ \text{Å}$$

Based on this radius, the distance between the cone apex and the anchor plane is given by

$$z_0 = \frac{r_{\text{sphere}}}{\tan\theta}$$

This relation ensures that the anchor sphere fits exactly inside the cone without intersecting the cone wall.

The height of the conical frustum is defined as the distance between the anchor plane and the solvent-facing mouth of the cone,

$$h = z_{\text{mouth}} - z_0$$

For all linker atoms located within the buried region ($z \leq z_{\text{mouth}}$), the linker must remain inside the cone wall. The cone mouth at $z_{\text{mouth}}$is assumed to lie within a flat surface representing the boundary between the pocket and bulk solvent. Linker atoms located above the mouth plane are therefore not allowed to intersect this surface.

Linker enumeration

The linker was represented as a chain of carbon-centered spheres. The first atom was placed along the z-axis away from the anchor atom. Subsequent spheres were added assuming tetrahedral bond geometry.

The following parameters were used to construct the chain:

Bond length

$$\text{d} = 1.54\ \text{Å}$$

Tetrahedral bond angle

$$\phi_{\text{tetra}} = 109.5°$$

Discrete torsional states

$$\phi \in \{0°, 120°, 240°\}$$

For each torsion vector, Cartesian coordinates were generated deterministically using the fixed bond length and tetrahedral bond angle.

Each linker atom was represented as a sphere with radius

$$r_{\text{sphere}} = 1.4\ \text{Å}$$

This radius provides a minimal steric exclusion volume that prevents unrealistic self-overlap while maintaining physically reasonable linker flexibility.

After each extension step, the newly introduced sphere was checked against the geometric constraints of the model:

Self-avoidance: Any two spheres separated by more than two bonds along the chain were required to have a minimum center-to-center distance of

$$2r_{\text{sphere}} = 2.8\ \text{Å}$$

Cone confinement: All spheres located within the buried region ($z \leq z_{\text{mouth}}$) were required to lie inside the cone. This condition was implemented by requiring the distance between the sphere surface and the cone wall to be positive.

Mouth-plane constraint: Spheres located above the mouth plane ($z \geq z_{\text{mouth}}$) were required not to intersect the planar boundary representing the transition to bulk solvent.

Because of the geometric constraints close to the anchor atom, the second sphere can intersect the cone wall for frustums with narrow cones. This effect arises from the tetrahedral bond angle combined with the finite bead radius. To avoid artificially excluding narrow cones that are otherwise physically accessible, the cone-confinement condition was therefore relaxed for the second atom.

Calculation of internal torsional strain

For each linker conformation, the torsional strain energy was estimated using a simple energetic model derived from the conformational preferences of alkane chains.

A penalty of

$$U_g = 0.8\ \text{kcal mol}^{-1}$$

was applied for torsions in gauche-like conformations relative to the trans state,

$$U_{\text{single}} = 0\ \text{kcal mol}^{-1}$$

In addition, an extra penalty of

$$U_{gg} = 0.6 \text{ kcal mol}^{-1}$$

was applied when two adjacent torsions were both in gauche-like states.

The torsional strain energy of conformation $k$was calculated as

$$E_k^{\text{tors}} = \sum_i U_g(\phi_i) + \sum_{(i,i+1)} U_{gg}(\phi_i, \phi_{i+1})$$

where $\phi_i$denotes the torsional state of bond i.

Removal of high-energy conformations

Only a subset of linker conformations is significantly populated under ambient conditions and therefore contributes meaningfully to the free energy changes associated with geometric confinement. High-energy conformations can therefore be removed, reducing computational cost while having minimal impact on the accuracy of the predictions.

The statistical weight of each conformation was calculated using a Boltzmann distribution based on the torsional strain energy:

$$p_k = \frac{\exp(-\beta E_k^{\text{tors}})}{\sum_j \exp(-\beta E_j^{\text{tors}})}$$

with

$$\beta = \frac{1}{RT}$$

where $R$is the gas constant and $T = 298.15$ K.

The conformational entropy associated with the discrete set of torsional states is given by

$$S_{\text{conf}} = -R \sum_k p_k \ln p_k$$

This quantity reflects the entropy arising from the distribution of population across distinct torsional basins.

For each conformation, the individual contribution to the conformational entropy is

$$s_k = -p_k \ln p_k$$

Conformations were ranked in descending order of s_k, and the smallest subset $S$was selected that satisfied

$$\sum_{k\in S} s_k \geq f \sum_{k} s_k$$

A value of

$$f = 0.8$$

was used for the calculations, meaning that the retained conformations account for at least 80% of the total conformational entropy of the system.

Exploration of the reduction of torsional freedom inside the frustum

Geometric confinement within the frustum restricts the range of torsional angles accessible to the linker relative to an unrestricted system.

In the present model, only torsional degrees of freedom were considered when estimating the entropy loss. Translational and rotational entropy contributions are expected to be largely independent of the detailed shape of the pocket and were therefore not included. Changes in vibrational entropy arising from bond stretching and angle bending are expected to be small at ambient temperature because these modes have significantly higher force constants than torsional rotations. While bond-angle flexibility can contribute to entropic penalties during ligand binding, its contribution is typically smaller than that of torsional degrees of freedom and was therefore neglected in the present model.

Each retained conformation $q_0$was treated as the center of a local torsional basin. The geometric restriction of continuous motion around this basin was quantified by probing the accessible region of torsional configuration space.

Because torsional motions in confined geometries are strongly coupled, the accessible configuration space was estimated using multidimensional directional probing in the full torsional coordinate space.

A unit vector $u$was sampled in the N_ϕ-dimensional space of torsional coordinates, and the maximum step length $\lambda(u)$was determined such that

$$q_0 + \lambda u$$

remained geometrically feasible.

For each sampled direction, the boundary distance $\lambda(u)$ was determined using a two-stage numerical search consisting of:

1. exponential bracketing to identify the approximate feasibility boundary
2. bisection refinement to locate the boundary with a specified tolerance

The standard parameters used for the directional probing were:

number of sampled directions

$$n_{\text{dirs}} = 100$$

initial step size: 5°

maximum search range: 90°

bisection tolerance: 0.1°

Sampling n_{dirs} directions produces a set of directional boundary distances

$$\{\lambda(u_1), \lambda(u_2), \ldots, \lambda(u_M)\}$$

where $M = n_{\text{dirs}}$. These boundary distances characterize the extent of the accessible region of torsional configuration space surrounding the reference conformation.

Conversion of torsional range into configuration-space volume

For a basin centered at q_0, the set of feasible nearby torsional displacements defines a bounded region in internal-coordinate space. The directional boundary distance $\lambda(u)$ measures how far this region extends along a direction u in torsional coordinate space.

If the feasible region were a perfect sphere with radius a, then $\lambda(u) = a$ for all directions u. If the region is anisotropic, $\lambda(u)$ depends on direction. The set of all directional distances therefore characterizes the local shape of the accessible configuration-space region.

The exact volume of an arbitrary high-dimensional region cannot be easily computed from directional boundary distances. Instead, a log-volume proxy was used based on the scaling relationship between volume and characteristic linear dimension.

For an internal-coordinate space with dimensionality n, the volume scales as a characteristic length raised to the n-th power. Accordingly, the effective local configuration-space volume was estimated from the directional distances as

$$\log V_{\text{eff}} \approx n \langle \log \lambda(u) \rangle_u$$

where

- $n = N_\phi$ is the number of torsional degrees of freedom
- $\langle \cdot \rangle_u$ denotes averaging over the sampled directions.

In practice, this was implemented as

$$\log V_{\text{eff}} = \frac{1}{M} \sum_{m=1}^{M} n \log \lambda(u_m)$$

where $M = n_{\text{dirs}}$ is the number of sampled directions.

This expression corresponds to using the geometric mean of the directional boundary distances as the characteristic size of the accessible torsional basin.

Reference torsional configuration-space volume

The local geometric volume must be compared to a reference volume corresponding to unconstrained torsional motion.

A reference torsional width of 90° (π/2 radians) was chosen because the model uses three canonical alkane torsional states at 0°, 120°, and 240°. The midpoint between neighboring states lies 60° from each minimum, so a width of 90° (±45°) approximates the natural basin around a torsional state before transitioning to the next one.

The reference configuration-space volume is therefore

$$V_{\text{ref}} = \left(\frac{\pi}{2}\right)^n$$

where n is the number of torsional degrees of freedom.

This provides a simple and internally consistent reference for comparing different geometries. More chemically specific reference widths could be introduced in future refinements. While the numerical magnitude of the calculated free-energy penalties depends on the chosen reference width, the qualitative trends and relative comparisons between different pocket geometries are largely independent of this choice.

Conversion of configuration-space volume into vibrational entropy

The local vibrational entropy associated with basin k was taken to be proportional to the logarithm of the accessible configuration-space volume:

$$\Delta S_k^{\text{vib}} = R\left(\log V_{\text{eff},k} - \log V_{\text{ref}}\right)$$

Because geometric confinement reduces the accessible torsional space,

$$\log\ V_{eff,k} < \log\ V_{ref}$$

and therefore

$$\Delta S_k^{vib} < 0$$

The corresponding free-energy penalty is

$$\Delta G_k^{vib} = -T\Delta S_k^{vib}$$

or explicitly

$$\Delta G_k^{vib} = -RT\left(\log\ V_{eff,k} - \log\ V_{ref}\right)$$

Substituting the volume proxy yields

$$\Delta G_k^{vib} = -RT\left(n\langle\log\ \lambda(u)\rangle_u - n\log\ \left(\frac{\pi}{2}\right)\right)$$

This quantity represents the coupled local vibrational free-energy penalty associated with geometric confinement around basin k.

Ensemble combination without double counting

The model distinguishes between:

1. entropy of distributing population across discrete torsional basins (i.e. Boltzmann weighing),

   and

2. local vibrational entropy within each basin.

These contributions must be combined without double counting.

If p_k are the torsion-only Boltzmann weights, then the ensemble vibrational free-energy contribution is

$$\Delta G_{\text{vib,total}} = -RT\ln\left(\sum_k p_k \exp\left[-\beta\Delta G_k^{\text{vib}}\right]\right)$$

This expresses how local geometric confinement changes the free energy of the pre-existing torsional ensemble.

The full free energy of the confined linker is obtained from

$$G_{\text{total}} = -RT\ln\left(\sum_k \exp\left[-\beta\left(E_k^{\text{tors}} + \Delta G_k^{\text{vib}}\right)\right]\right)$$

This combines:

- intrinsic torsional strain of each basin,
- local vibrational penalty caused by confinement.

Because both enter inside the partition sum, this formulation avoids double counting conformational entropy.

Normalization of Entropy penalty

To remove residual geometric artifacts of the cone representation, all calculated entropy penalties were referenced to a nearly unrestricted system consisting of a cone with half-angle 89° and negligible height (0.1 Å). This reference system represents an effectively unconstrained environment and ensures that the reported free-energy penalties reflect only the additional confinement imposed by narrower or deeper cones.

Conversion to binding-affinity changes

Free-energy penalties were converted into fold-changes in dissociation constant via

$$\frac{K_d^{\text{confined}}}{K_d^{\text{reference}}} = \exp\left(\frac{\Delta G}{RT}\right)$$

At 298 K, $RT \approx 0.593$kcal/mol, so even modest free-energy penalties produce large effects on affinity.

Table S1. Information on co-crystal structure data used to determine linkerability of DEL-hits at linker attachment site (Figure 7d).

PDB ID	Ligand ID	Atom ID	Linkerability
4COD	KV1	C1	unrestricted
4X8G	3Z0	C23	intermediate
4Z2B	4LC	C01	inaccessible
5HX6	65U	C09	inaccessible
5KU9	6XJ	C	unrestricted
5LAR	6SH	N	inaccessible
5U9D	83P	C1	unrestricted
6FEW	D6W	C33	restricted
6UVK	QHY	O1	intermediate
6W35	SKV	C31	inaccessible
6W44	SLJ	C9	inaccessible
6W45	SLG	C	intermediate
6W4C	SL7	C1	unrestricted
6WFG	U3V	C9	unrestricted
6WFK	U2J	C9	unrestricted
6WFN	U2J	C9	unrestricted
6WFO	U3Y	C9	unrestricted
7AW2	S4W	C	restricted
7AW3	S4Z	C	unrestricted
7AW4	S5E	C21	unrestricted
7JT7	TG3	C01	unrestricted
7L9A	XWP	C1	unrestricted
7LTN	YCV	C17	unrestricted
7NTH	URW	C24	unrestricted
7NTI	UWZ	C19	unrestricted
7SS8	C0C	C01	unrestricted
7U3J	L6U	C01	unrestricted
7U3L	L4X	C38	unrestricted
7UBO	MJN	C01	unrestricted
7URB	O5O	C4	unrestricted
8AHE	M2U	C1	unrestricted
8AHF	M3U	C1	unrestricted
8AHG	M4I	C1	restricted
8AHH	M4X	C1	restricted
8AHI	M56	C1	restricted
8ANS	MDI	C11	inaccessible
8RZB	A1H4A	C8	unrestricted
8UDF	WB0	C1	unrestricted
8UDJ	WB5	C1	unrestricted
9BJK	A1APU	C01	unrestricted
9E9H	A1BH6	C8	intermediate

9FOC	A1IFS	C8	inaccessible
9FOE	A1IEF	C11	unrestricted
9G7H	A1IIZ	C17	inaccessible
9GIJ	A1IL0	C37	inaccessible
9HGM	A1IUL	C01	unrestricted
9OG3	A1CA4	C1	unrestricted
9S0O	A1JKT	C	unrestricted

Table S2. Information on co-crystal structure data used to determine linkerability of protein ligands for PROTAC development at linker attachment site (Figure 7e).

PDB ID	Ligand ID	Atom ID	Linkerability	Reference PROTAC
4BJX	73B	03	Unrestricted	[2]
5WMD	6JE	O31	Unrestricted	[3]
7KBS	RAL	C24	Restricted	[4]
3ERT	OHT	C25	Intermediate	[5]
4RV6	RPB	N3	Inaccessible	[6]
4R6E	3JD	NAP	Inaccessible	[7]
9ETQ	A1H63	C18	Inaccessible	[8]
4XLI	1N1	C21	Unrestricted	[9]
4WKQ	IRE	CAN	Unrestricted	[10]
4ZAU	YY3	C12	Unrestricted	[11]
5X2K	0UN	CAB	Unrestricted	[12]
5P9J	8E8	CAD	Unrestricted	[13]
5P9G	7G6	C43	Unrestricted	[14]
2XP2	VGH	N25	Unrestricted	[15]
4MKC	4MK	N11	Unrestricted	[16]
6MX8	6GY	C28	Unrestricted	[17]
4ASD	BAX	C31	Intermediate	[18]
5L2I	LQQ	N07	Unrestricted	[19]
5L2T	6ZZ	N30	Unrestricted	[20]
5D1J	56H	N25	Unrestricted	[21]
4QNQ	1XJ	C39	Unrestricted	[22]
5G0J	N4R	C	Unrestricted	[23]
6THV	N9W	C17	Unrestricted	[24]
8OWZ	KZU	C29	Intermediate	[25]
6USZ	QH4	C25	Unrestricted	[26]
6OIM	MOV	O3	Inaccessible	[27]
4R1V	3E8	C35	Unrestricted	[28]
6VGL	RXT	CAE	Inaccessible	[29]
4XUF	P30	C37	Unrestricted	[14]
3UE4	DB8	CAO	Unrestricted	[14]
2GVJ	DGB	CAE	Inaccessible	[30]
7L9Y	XRM	N22	Inaccessible	[31]

References

1. Ge J, Li S, Weng G, Wang H, Fang M, Sun H, Deng Y, Hsieh CY, Li D, Hou T. PROTAC-DB 3.0: an updated database of PROTACs with extended pharmacokinetic parameters. Nucleic Acids Res. 2025;53(D1):D1510-D5. doi: 10.1093/nar/gkae768. PubMed PMID: 39225044; PMCID: PMC11701630.

2. Chan KH, Zengerle M, Testa A, Ciulli A. Impact of Target Warhead and Linkage Vector on Inducing Protein Degradation: Comparison of Bromodomain and Extra-Terminal (BET) Degraders Derived from Triazolodiazepine (JQ1) and Tetrahydroquinoline (I-BET726) BET Inhibitor Scaffolds. J Med Chem. 2018;61(2):504-13. Epub 20170622. doi: 10.1021/acs.jmedchem.6b01912. PubMed PMID: 28595007; PMCID: PMC5788402.

3. Lu J, Qian Y, Altieri M, Dong H, Wang J, Raina K, Hines J, Winkler JD, Crew AP, Coleman K, Crews CM. Hijacking the E3 Ubiquitin Ligase Cereblon to Efficiently Target BRD4. Chem Biol. 2015;22(6):755-63. Epub 20150604. doi: 10.1016/j.chembiol.2015.05.009. PubMed PMID: 26051217; PMCID: PMC4475452.

4. Hu J, Hu B, Wang M, Xu F, Miao B, Yang CY, Wang M, Liu Z, Hayes DF, Chinnaswamy K, Delproposto J, Stuckey J, Wang S. Discovery of ERD-308 as a Highly Potent Proteolysis Targeting Chimera (PROTAC) Degrader of Estrogen Receptor (ER). J Med Chem. 2019;62(3):1420-42. Epub 20190118. doi: 10.1021/acs.jmedchem.8b01572. PubMed PMID: 30990042.

5. Ohoka N, Okuhira K, Ito M, Nagai K, Shibata N, Hattori T, Ujikawa O, Shimokawa K, Sano O, Koyama R, Fujita H, Teratani M, Matsumoto H, Imaeda Y, Nara H, Cho N, Naito M. In Vivo Knockdown of Pathogenic Proteins via Specific and Nongenetic Inhibitor of Apoptosis Protein (IAP)-dependent Protein Erasers (SNIPERs). J Biol Chem. 2017;292(11):4556-70. Epub 20170202. doi: 10.1074/jbc.M116.768853. PubMed PMID: 28154167; PMCID: PMC5377772.

6. Wang S, Han L, Han J, Li P, Ding Q, Zhang QJ, Liu ZP, Chen C, Yu Y. Uncoupling of PARP1 trapping and inhibition using selective PARP1 degradation. Nat Chem Biol. 2019;15(12):1223-31. Epub 20191028. doi: 10.1038/s41589-019-0379-2. PubMed PMID: 31659317; PMCID: PMC6864272.

7. Li G, Lin SS, Yu ZL, Wu XH, Liu JW, Tu GH, Liu QY, Tang YL, Jiang QN, Xu JH, Huang QL, Wu LX. A PARP1 PROTAC as a novel strategy against PARP inhibitor resistance via promotion of ferroptosis in p53-positive breast cancer. Biochem Pharmacol. 2022;206:115329. Epub 20221027. doi: 10.1016/j.bcp.2022.115329. PubMed PMID: 36309080.

8. Li Y, Chen L, He T, Geng P, Yao H, Wang H, Fang L, Hu G, Tang P, Yu Y, Zhang C, Yan P, inventors; Haisco Pharmaceutical Group Co Ltd, assignee. PARP-1 Degradation Agent and Use Thereof2023 April 27, 2023.

9. Mao W, Vandecan NM, Bingham CR, Tsang PK, Ulintz P, Sexton R, Bochar DA, Merajver SD, Soellner MB. Selective and Potent PROTAC Degraders of c-Src Kinase. ACS Chem Biol.

2024;19(1):110-6. Epub 20231219. doi: 10.1021/acschembio.3c00548. PubMed PMID: 38113191; PMCID: PMC11776100.

10. Burslem GM, Smith BE, Lai AC, Jaime-Figueroa S, McQuaid DC, Bondeson DP, Toure M, Dong H, Qian Y, Wang J, Crew AP, Hines J, Crews CM. The Advantages of Targeted Protein Degradation Over Inhibition: An RTK Case Study. Cell Chem Biol. 2018;25(1):67-77 e3. Epub 20171109. doi: 10.1016/j.chembiol.2017.09.009. PubMed PMID: 29129716; PMCID: PMC5831399.

11. He K, Zhang Z, Wang W, Zheng X, Wang X, Zhang X. Discovery and biological evaluation of proteolysis targeting chimeras (PROTACs) as an EGFR degraders based on osimertinib and lenalidomide. Bioorg Med Chem Lett. 2020;30(12):127167. Epub 20200404. doi: 10.1016/j.bmcl.2020.127167. PubMed PMID: 32317208.

12. Hu W, He J, Xie T, Zhou M, Liu M, Wang X. Discovery of potent CRBN-recruiting epidermal growth factor receptor (EGFR) degraders in vitro. Invest New Drugs. 2025;43(3):560-81. Epub 20250429. doi: 10.1007/s10637-025-01539-2. PubMed PMID: 40301162.

13. Yang Z, Sun Y, Ni Z, Yang C, Tong Y, Liu Y, Li H, Rao Y. Merging PROTAC and molecular glue for degrading BTK and GSPT1 proteins concurrently. Cell Res. 2021;31(12):1315-8. Epub 20210820. doi: 10.1038/s41422-021-00533-6. PubMed PMID: 34417569; PMCID: PMC8648895.

14. Huang HT, Dobrovolsky D, Paulk J, Yang G, Weisberg EL, Doctor ZM, Buckley DL, Cho JH, Ko E, Jang J, Shi K, Choi HG, Griffin JD, Li Y, Treon SP, Fischer ES, Bradner JE, Tan L, Gray NS. A Chemoproteomic Approach to Query the Degradable Kinome Using a Multi-kinase Degrader. Cell Chem Biol. 2018;25(1):88-99 e6. Epub 20171109. doi: 10.1016/j.chembiol.2017.10.005. PubMed PMID: 29129717; PMCID: PMC6427047.

15. Chen JJ, Jin JM, Gu WJ, Zhao Z, Yuan H, Zhou YD, Nagle DG, Xi QL, Zhang XM, Sun QY, Wu Y, Zhang WD, Luan X. Crizotinib-based proteolysis targeting chimera suppresses gastric cancer by promoting MET degradation. Cancer Sci. 2023;114(5):1958-71. Epub 20230207. doi: 10.1111/cas.15733. PubMed PMID: 36692137; PMCID: PMC10154821.

16. Zhou H, Hu M, Jie H, Li Y, Tang K, Pan L, Liu C, Liu Z, Chen W, Chen Y, Luo Y, Gong Y, Xie Y. Discovery of orally bioavailable ALK PROTACs based ceritinib against ALK positive cancers. Eur J Med Chem. 2024;279:116827. Epub 20240912. doi: 10.1016/j.ejmech.2024.116827. PubMed PMID: 39288596.

17. Ren C, Sun N, Liu H, Kong Y, Sun R, Qiu X, Chen J, Li Y, Zhang J, Zhou Y, Zhong H, Yin Q, Song X, Yang X, Jiang B. Discovery of a Brigatinib Degrader SIAIS164018 with Destroying Metastasis-Related Oncoproteins and a Reshuffling Kinome Profile. J Med Chem. 2021;64(13):9152-65. Epub 20210617. doi: 10.1021/acs.jmedchem.1c00373. PubMed PMID: 34138566.

18. Si R, Hai P, Zheng Y, Wang J, Zhang Q, Li Y, Pan X, Zhang J. Discovery of intracellular self-assembly protein degraders driven by tumor-specific activatable bioorthogonal reaction. Eur J Med Chem. 2023;257:115497. Epub 20230516. doi: 10.1016/j.ejmech.2023.115497. PubMed PMID: 37216813.

19. Rana S, Bendjennat M, Kour S, King HM, Kizhake S, Zahid M, Natarajan A. Selective degradation of CDK6 by a palbociclib based PROTAC. Bioorg Med Chem Lett. 2019;29(11):1375-9. Epub 20190326. doi: 10.1016/j.bmcl.2019.03.035. PubMed PMID: 30935795; PMCID: PMC6487213.

20. Pu C, Liu Y, Deng R, Xu Q, Wang S, Zhang H, Luo D, Ma X, Tong Y, Li R. Development of PROTAC degrader probe of CDK4/6 based on DCAF16. Bioorg Chem. 2023;138:106637. Epub 20230527. doi: 10.1016/j.bioorg.2023.106637. PubMed PMID: 37276679.

21. Olson CM, Jiang B, Erb MA, Liang Y, Doctor ZM, Zhang Z, Zhang T, Kwiatkowski N, Boukhali M, Green JL, Haas W, Nomanbhoy T, Fischer ES, Young RA, Bradner JE, Winter GE, Gray NS. Pharmacological perturbation of CDK9 using selective CDK9 inhibition or degradation. Nat Chem Biol. 2018;14(2):163-70. Epub 20171218. doi: 10.1038/nchembio.2538. PubMed PMID: 29251720; PMCID: PMC5912898.

22. Khan S, Cao L, Wiegand J, Zhang P, Zajac-Kaye M, Kaye FJ, Zheng G, Zhou D. PROTAC-Mediated Dual Degradation of BCL-xL and BCL-2 Is a Highly Effective Therapeutic Strategy in Small-Cell Lung Cancer. Cells. 2024;13(6). Epub 20240317. doi: 10.3390/cells13060528. PubMed PMID: 38534371; PMCID: PMC10968744.

23. An Z, Lv W, Su S, Wu W, Rao Y. Developing potent PROTACs tools for selective degradation of HDAC6 protein. Protein Cell. 2019;10(8):606-9. doi: 10.1007/s13238-018-0602-z. PubMed PMID: 30603959; PMCID: PMC6626596.

24. Zhai S, Honin I, Schäker-Hübner L, Hanl M, Jacobi L, Dressler F, Pieńkowska DE, König P, Gerhartz J, Voget R, Bendas G, Gütschow M, Meissner F, Burckhardt B, Nowak RP, Steinebach C, Hansen FK. Development and characterization of the first selective class IIb histone deacetylase degraders. ChemRXIv. 2025.

25. Schiedel M, Herp D, Hammelmann S, Swyter S, Lehotzky A, Robaa D, Olah J, Ovadi J, Sippl W, Jung M. Chemically Induced Degradation of Sirtuin 2 (Sirt2) by a Proteolysis Targeting Chimera (PROTAC) Based on Sirtuin Rearranging Ligands (SirReals). J Med Chem. 2018;61(2):482-91. Epub 20170417. doi: 10.1021/acs.jmedchem.6b01872. PubMed PMID: 28379698.

26. Yang F, Wen Y, Wang C, Zhou Y, Zhou Y, Zhang ZM, Liu T, Lu X. Efficient targeted oncogenic KRAS(G12C) degradation via first reversible-covalent PROTAC. Eur J Med Chem. 2022;230:114088. Epub 20220103. doi: 10.1016/j.ejmech.2021.114088. PubMed PMID: 35007863.

27. Yang N, Fan Z, Sun S, Hu X, Mao Y, Jia C, Cai X, Xu T, Li B, Li Y, Han L, Wei T, Qian X, Qin W, Li P, Zheng Z, Li S. Discovery of highly potent and selective KRAS(G12C) degraders by VHL-

recruiting PROTACs for the treatment of tumors with KRAS(G12C)-Mutation. Eur J Med Chem. 2023;261:115857. Epub 20231013. doi: 10.1016/j.ejmech.2023.115857. PubMed PMID: 37852032.

28. Li P, Jia C, Fan Z, Hu X, Zhang W, Liu K, Sun S, Guo H, Yang N, Zhu M, Zhuang X, Xiao J, Zheng Z, Li S. Discovery of novel exceptionally potent and orally active c-MET PROTACs for the treatment of tumors with MET alterations. Acta Pharm Sin B. 2023;13(6):2715-35. Epub 20230119. doi: 10.1016/j.apsb.2023.01.014. PubMed PMID: 37425039; PMCID: PMC10326257.

29. Kargbo RB. PROTAC-Mediated Degradation of Janus Kinase as a Therapeutic Strategy for Cancer and Rheumatoid Arthritis. ACS Med Chem Lett. 2021;12(6):945-6. Epub 20210510. doi: 10.1021/acsmedchemlett.1c00245. PubMed PMID: 34141075; PMCID: PMC8201481.

30. Zhu X, Liu H, Chen L, Wu C, Liu X, Cang Y, Jiang B, Yang X, Fan G. Addressing the Enzyme-independent tumor-promoting function of NAMPT via PROTAC-mediated degradation. Cell Chem Biol. 2022;29(11):1616-29 e12. Epub 20221101. doi: 10.1016/j.chembiol.2022.10.007. PubMed PMID: 36323324.

31. Wigle TJ, Ren Y, Molina JR, Blackwell DJ, Schenkel LB, Swinger KK, Kuplast-Barr K, Majer CR, Church WD, Lu AZ, Mo J, Abo R, Cheung A, Dorsey BW, Niepel M, Perl NR, Vasbinder MM, Keilhack H, Kuntz KW. Targeted Degradation of PARP14 Using a Heterobifunctional Small Molecule. Chembiochem. 2021;22(12):2107-10. Epub 20210504. doi: 10.1002/cbic.202100047. PubMed PMID: 33838082.
